# Viral load-guided immunosuppression after lung transplantation (VIGILung)—study protocol for a randomized controlled trial

**DOI:** 10.1186/s13063-020-04985-w

**Published:** 2021-01-11

**Authors:** Jens Gottlieb, Alexander Reuss, Konstantin Mayer, Karin Weide, Carmen Schade-Brittinger, Susanne Hoyer, Peter Jaksch

**Affiliations:** 1 Department of Respiratory Medicine OE6870, Hannover Medical School, 30625 Hannover, Germany; 2 BREATH (Biomedical Research in End-stage and obstructive Lung Disease Hannover), Hannover, Germany; 3 Coordinating Centre for Clinical Trials Marburg (KKS Marburg), Philipps-University Marburg, Marburg, Germany; 4 University of Giessen and Marburg Lung Center (UGMLC), University Hospital Giessen, Justus Liebig University of Giessen, Giessen, Germany; 5 Division of Thoracic Surgery, Medical University of Vienna, Vienna, Austria

**Keywords:** Lung transplantation, Immunosuppression, Graft rejection, Kidney failure, Torque teno virus, Randomized controlled trial

## Abstract

**Background:**

Immunosuppression including high-dose calcineurin inhibitors (CNI) is essential after lung transplantation. Dosing is usually guided by therapeutic drug monitoring adjusted to target trough levels of CNIs to keep the balance between over-dose causing severe toxicity and increased risk of infections or under-dose with a risk of graft injury. Adaptation of CNI-based immunosuppression by monitoring of torque teno virus (TTV), a latent nonpathogenic DNA virus, measured in the whole blood in addition to conventional therapeutic drug monitoring may reduce the toxicity of immunosuppression with similar efficacy.

**Methods/design:**

An open-label, randomized, controlled, parallel-group, multicenter trial in lung transplant recipients will be conducted to investigate the safety and efficacy of immunosuppression guided by TTV monitoring as an add-on to conventional therapeutic drug monitoring. Adult lung transplant recipients 21 to 42 days after transplantation are eligible to participate. Patients (*N* = 144) will be randomized 1:1 to the experimental intervention (arm 1: immunosuppression guided by TTV monitoring in addition to conventional therapeutic drug monitoring of tacrolimus trough levels) and control intervention (arm 2: conventional therapeutic drug monitoring). Outcomes will be assessed 12 months after randomization with the change in glomerular filtration rate as the primary endpoint. Secondary endpoints will be additional measurements of renal function, allograft function, incidence of acute rejections, incidence of chronic lung allograft dysfunction, graft loss, and infections.

**Discussion:**

The results of this randomized controlled trial may reduce the toxicity of immunosuppression after lung transplantation while maintaining the efficacy of immunosuppression. Study results are transferable to all other solid organ transplantations.

**Trial registration:**

ClinicalTrials.gov NCT04198506. Registered on 12 December 2019

## Background

High-dose immunosuppression containing calcineurin inhibitors (CNI) is essential after lung transplantation. Dosing is usually guided by fixed target levels, established to keep the balance between over-dose causing toxicity and increased risk of infections or under-dose with a risk of graft injury.

Therapeutic drug monitoring represents the current gold standard of guiding immunosuppression after solid organ transplantation [1]. Despite rigorous monitoring, acute rejection occurs in approximately one third of patients in the first year after lung transplantation [2]. Immunosuppressive regimens are responsible for considerable toxicity. Approximately 24% of the recipients will develop kidney failure within 1 year of transplantation and 2% end-stage kidney disease. Increased infection rates are associated with over-immunosuppression, and infections are the leading cause of death during the first postoperative year.

Clinical experience suggests that individual tailoring of immunosuppression could potentially optimize patient outcome [3–6]. Reliable, reproducible, cost-effective, and non-invasive biomarkers are needed to reduce the risk of graft injury and toxicity to guide immunosuppression.

Latent DNA viruses in the whole blood (e.g., torque teno virus (TTV)) are detectable in the vast majority of lung transplant recipients [3].The load of these latent viruses was associated with the strength of immunosuppression in solid organ recipients [7].

DNA viruses in the whole blood (torque teno virus/TTV, HHV-6, EBV) and urine (BK virus) can be detected in the majority of humans including transplant recipients. Some of them do not cause symptoms of infection, for example, TTV. The viral load of TTV was used as a surrogate biomarker of cell-mediated immunity (load increasing with the strength of immunosuppression) but has never been studied in a prospective trial. It is suggested by pivotal studies [3] that CNI-based immunosuppression may be reduced in the majority of lung transplant recipients during the first postoperative year.

Guiding immunosuppression by an immune response assay in a prospective trial resulted in reductions in CNI doses of 13–25% within the first year after liver transplantation, with documented reductions in bacterial and fungal infections [6]. In lung transplant recipients, a CNI reduction of 50% has led to an improvement of GFR (CKD-EPI) by 10 ml/min/1.73 m^2^ after 12 months in a recent randomized trial [8]. With the management of trough levels by TTV load, it is expected that a similar CNI dose reduction can be achieved.

The results of the VIGILung trial may have an impact on therapeutic strategies for patients after lung transplantation. Study results may be transferable to all other solid organ transplantations.

## Methods/design

### Objectives

The aim of this randomized controlled trial is the prospective investigation of the safety and efficacy of an individual adaptation of the tacrolimus-based immunosuppression by a non-invasive biomarker (torque teno virus (TTV) load in the whole blood).

The primary endpoint will be Δ-glomerular filtration rate (GFR) defined as the change of the glomerular filtration rate GFR between randomization and 12 months thereafter as an indicator for toxicity. GFR will be estimated using the Chronic Kidney Disease Epidemiology Collaboration (CKD-EPI) formula [9].

Secondary outcomes include further parameters on renal function, parameters regarding lung allograft function forced expiratory volume in 1 s (FEV1) in % of baseline, incidence of acute rejections, lymphocytic bronchiolitis and/or chronic lung allograft dysfunction, re-transplantation or death due to graft failure, infections (cytomegalovirus (CMV), community-acquired respiratory viruses (CARV), fungal or bacterial infections), incidence of unscheduled emergency hospitalizations or admissions for intensive care unit (ICU), Quality of Life Questionnaire (EQ-5D) and exercise capacity, the state of immune-system (cluster of differentiation 4 (CD4)-lymphocytes, donor-specific antibodies (DSA), IgG level and use of rescue immune therapy), and details on the tacrolimus immunosuppression (tacrolimus trough levels, tacrolimus doses, and number of adjustment of immunosuppression).

## Design and participants

The screening of the patients will be performed in two participating study sites (Hannover Medical School and University of Vienna). We assume that screening of 250 patients will result in 144 subjects eligible for the study. The recruitment period is expected to be 39 months.

Selected in- and exclusion criteria are as follows:

Inclusion criteria:

- Adult patients 21 to 42 days after de novo lung transplantation (bilateral or combined)
- Tacrolimus based immunosuppression
- Detectable TTV load at randomization (> 2.7 log 10 copies/mL)
- Women of child-bearing potential: negative serum pregnancy test and highly effective methods of contraception throughout the study.

Exclusion criteria:

- History or high-risk of obstructive airway complications after lung transplantation
- Respiratory failure (need for oxygen therapy or ventilation at screening) inability to undergo transbronchial biopsy
- Advanced kidney failure (GFR CKD-EPI < 30 ml/min/1.73 m^2^ at inclusion and/or current renal replacement therapy at inclusion or randomization
- Advanced liver cirrhosis (CHILD-Pugh Score C) after lung transplantation
- Fluctuating tacrolimus drug levels (less than 20% in target range after transplantation)
- Symptoms of significant mental illness with the inability to cooperate or communicate with the investigator
- Unlikeliness to comply with the study requirements
- HIV positivity

### Randomization

Randomization will be performed centrally via telefax by the Center for Clinical Trials (KKS) of the Philipps-University Marburg for patients matching all eligibility criteria and will be stratified by high-risk CMV status (D+R−) (yes/no) and center using randomization lists generated at KKS with permuted blocks of randomly varying size. The chance for allocation to the control and experimental group is 1:1. Investigators at the participating sites request the randomization result by faxing the completed randomization form, whereupon KKS staff will consult the randomization database and transmit the result to the investigator.

### Trial intervention and control

After lung transplantation (day 0), all patients will receive a standard tacrolimus-based triple immunosuppressive regime (tacrolimus, prednisolone, and a cell cycle inhibitor (depending on induction therapy with alemtuzumab)). Tacrolimus-based immunosuppressive protocols represent the standard of care worldwide [2] and were therefore chosen as the comparator. Inclusion and screening will occur 21 to 42 days after lung transplantation; a further screening visit will occur 4 weeks later, especially to evaluate the achievement of a sufficient TTV level.

### Follow-up protocol

**Fig. 1:**
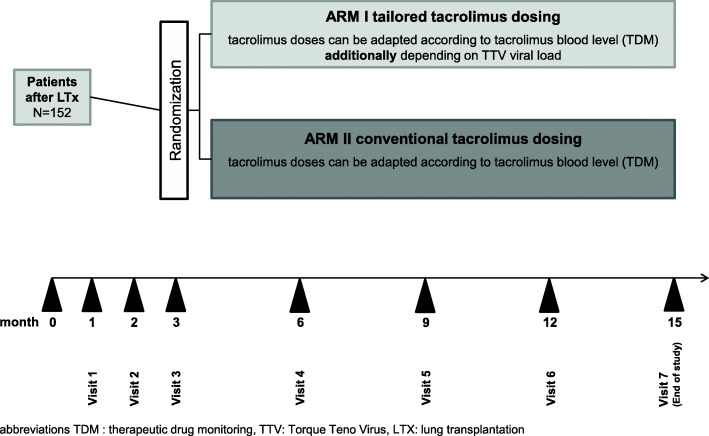
Trial flow chart and treatment groups

At visit 3 patients will be assigned to one of the two treatment groups detailed in Fig. 1.

Study visits will be performed at months 1, 2, and 3 after transplantation and at months 3, 6, 9, and 12 after randomization. The final examination will be performed at month 12 after randomization or in case of a premature end of study treatment.

In both treatment arms, during maintenance, immunosuppression interruption of one out of three immunosuppressive components for up to 4 weeks will be allowed, e.g., in case of cell cycle inhibitors induce severe toxicity (e.g., leukopenia < 4000/μl, thrombocytopenia < 50,000/μl, anemia with hemoglobin < 8 g/dl).

After transplantation, tacrolimus and additional immunosuppressive drugs, usually a cell cycle inhibitor (mycophenolate mofetil, mycophenolate sodium, azathioprine) and prednisolone, are given.

In case of induction therapy with alemtuzumab, tacrolimus and prednisolone are given from day 0 and cell cycle inhibitors are delayed until the restoration of lymphocyte count—usually after 12 months [5, 10].

Adjustment of tacrolimus immunosuppression will be established in predefined steps, derived from previous trials in immunosuppression [11]. Immunosuppression (IS) will be managed according to predefined steps (with gradually reduced tacrolimus target levels from step 8 to 1. Patients without induction with alemtuzumab will start on step 6 (target trough level of tacrolimus 8–12 ng/ml), and patients with induction with alemtuzumab will start on step 4 (target trough level of tacrolimus 6–10 ng/ml) in this scheme. Trough levels of tacrolimus will be determined according to liquid chromatography coupled with mass spectrometry (LC-MS) at least once a month.

All patients who experience an acute cellular rejection after reduction of CNI will be put on increased doses of calcineurin inhibitors.

In arm II, the tacrolimus-based immunosuppression will be guided by conventional therapeutic drug monitoring (TDM). In patients randomized to the conventional CNI dosing, the immunosuppressive drugs should be applied according to trough levels, signs of toxicity, and center practice.

**Fig. 2:**
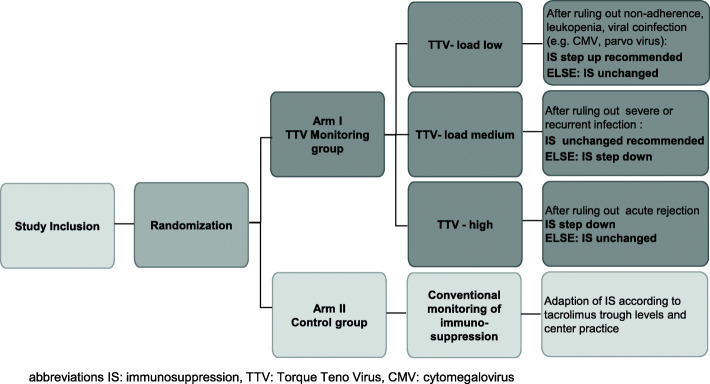
Adaptation of immunosuppression in the treatment groups

In arm I, the immunosuppression will be guided by the torque teno virus (TTV) monitoring and conventional therapeutic drug monitoring (TDM) (Fig. 2). Reduction in target trough levels will only be performed in stable patients after exclusion of silent rejection in the transbronchial biopsy.

The TTV load reached after TTV stabilization (expected after 3 months) will be used to guide immunosuppression, which will be tailored according to the scheme above (Fig. 2).

Maintenance immunosuppression will be guided by therapeutic drug monitoring (TDM) of tacrolimus trough levels. Furthermore, signs of toxicity of tacrolimus and cell cycle inhibitors (e.g., leukopenia, gastrointestinal side effects, hypogammaglobulinemia) may lead to dose adaptation and switch between various cell cycle inhibitors.

### Measurements

Most important measurement is creatinine (from blood chemistry) as the key parameter to determine the primary endpoint ΔGFR between randomization and 12 months thereafter.

Another key measurement within this trial is the TTV load in arm 1 patients. TTV load is classified as low with TTV load < 7 log_10_, as medium with TTV load from 7 to 9.5 log_10_, and as high with a TTV load of > 9.5 log_10._

TTV DNA quantitation will be done by TaqMan real-time PCR using probe and primers as described previously [12]. The linear range of TTV quantitation ranges from 2.7 to 10.7 log10 copies/mL as determined by the use of 10-fold dilutions of a plasmid standard. The limit of detection in plasma is 2.7 log10 copies/ml. In each run, a TTV DNA standard and positive and negative controls will be included and any signs of PCR inhibition will be assessed by quantitation of the known amount of control DNA spiked into the samples before DNA extraction [12].

### Outcome assessments

For the primary endpoint ΔGFR between randomization and 12 months thereafter, creatinine from the serum will be measured to calculate GFR (CKD-EPI) at each visit.

The main secondary outcomes are assessed as follows:

- GFR (CKD-EPI) at 1 and 2 months after transplantation (screening visits) and 0, 3, 6, 9, and 12 months after randomization
- GFR (cystatin) at randomization and 12 months after randomization
- Proportion of patients with biopsy-proven acute cellular rejection (grade A1 or higher) within 12 months after randomization
- Proportion of patients with an episode of biopsy-proven lymphocytic bronchitis (grade B1R or higher) within 12 months after randomization
- Proportion of patients with cytomegalovirus (CMV) infection and number of CMV disease episodes within 12 months after randomization
- Proportion of patients with community-acquired respiratory viral infection (CARV) within 12 months after randomization
- Proportion of patients with fungal and bacterial infections within 12 months after randomization
- Proportion of patients with any of the abovementioned infections within 12 months after randomization
- Proportion of patients with unscheduled or emergency hospitalizations after randomization
- Proportion of patients with ICU admissions after randomization
- Quality of life (EQ-5D visual analog scale) at screening visits and 0, 3, 6, 9, and 12 months after randomization
- Proportion of patients with new or progressive malignancy within 12 months after randomization
- Tacrolimus trough levels at screening visits and 0, 3, 6, 9, and 12 months after randomization
- Daily tacrolimus dose [mg] at screening visits and 0, 3, 6, 9, and 12 months after randomization
- Proportion patients with increased/unchanged/decreased (compared to the previous visit) target trough levels of tacrolimus at screening visits and 0, 3, 6, 9, and 12 months after randomization
- Exercise capacity measured by the percent predicted distance achieved in the 6-min walk test at randomization and 12 months thereafter
- CD4 lymphocyte count at 0, 6, and 12 months after randomization
- Proportion of patients with presence of donor-specific antibodies at 0, 6, and 12 months after randomization
- FEV1 in % baseline value at screening visits and 0, 3, 6, 9, and 12 months after randomization
- Incidence of chronic lung allograft dysfunction between randomization and 12 months thereafter
- IgG level at 0, 6, and 12 months after randomization
- Proportion of patients with rescue immunotherapy (defined by the use of ATG, rituximab, alemtuzumab, plasma exchange, immunoadsorption) between randomization and 12 months thereafter

**Fig. 3:**
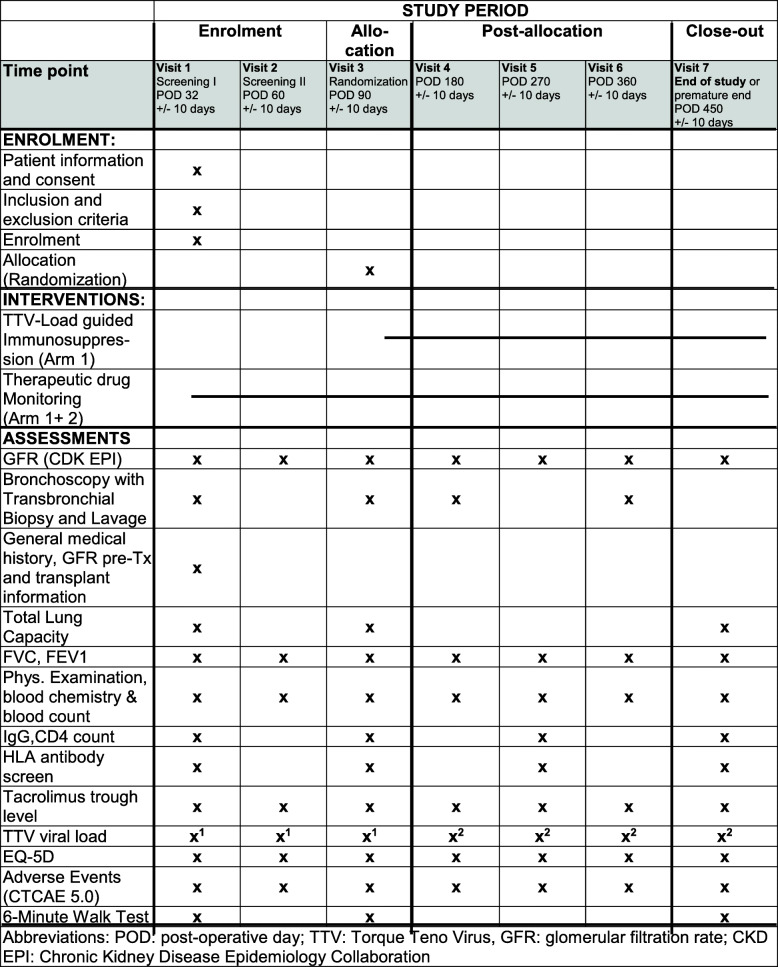
Data collection time points (SPIRIT figure)

Data collection time points are displayed in Fig. 3.

### Statistical analysis

Unblinding procedures are not applicable because the trial is not blinded. Data analysts will be blinded for the randomization results until database closure for the final analysis. Apart from safety reports to the Data and Safety Monitoring Committee (DSMC), no interim analyses are planned.

#### Primary efficacy analysis

The primary efficacy endpoint ΔGFR is defined as the change of the glomerular filtration rate GFR between randomization and 12 months thereafter. GFR will be estimated using the CKD-EPI formula. The primary endpoint analysis will be adjusted for randomization strata and baseline. No subgroup analyses are planned.

An analysis of covariance (ANCOVA) with strata used at randomization and treatment as fixed factors, and the baseline GFR as a covariate is used to test for a difference in location of ΔGFR (in ml/min/1.73 m^2^) between the groups (two-sided at a 0.05 significance level). The analysis of the primary endpoint will be based on the intention-to-treat population.

#### Secondary endpoints

Categorical variables will be reported as absolute and relative frequency, continuous variables by median, mean, standard deviation, 95% confidence interval, minimum, and maximum. Student’s *t* test, analysis of covariance or their non-parametric analogs, as well as chi-square or Fisher’s exact test, furthermore (generalized) linear mixed models will be applied as appropriate for the types of variables, group, high-risk CMV status, and time, as well as a group-by-time interaction term will be used as covariates in the mixed models. All statistical tests will be carried out at a two-sided significance level of 5% without adjustment for multiple comparisons. Full details will be stated in a statistical analysis plan.

#### Safety analyses

The as-treated population is defined as all included patients who have undergone at least one blood sample for therapy monitoring after randomization. Analysis of safety will be based on the as-treated population, i.e., patients will be analyzed according to the intervention they actually received.

#### Sample size calculation

The sample size calculation is performed assuming a *t* test of ΔGFR. Because adjusting for stratification factors is assumed to reduce variability, the power for the ANCOVA test of the treatment variable is expected to be rather higher than lower compared to the *t* test.

The sample size calculation is based on the assumption of a mean decline in GFR of 15 between randomization and 12 months thereafter and a standard deviation of 20 in the control group. Two randomized trials [8, 13] observed effect sizes of a similar magnitude. Compared to the control group, a mean decline in GFR of 5 in the experimental group (i.e., difference of 10 in GFR declines between the intervention groups) is regarded as clinically relevant. For a 2-sided significance level of 5% and a power of 80%, 64 patients per group (*n* = 128 in total) are needed to achieve a power of 80% if the true difference in means is 10 and the standard deviation is 20 in both. With an estimated dropout rate of 10%, 144 patients have to be randomized.

#### Data collection

Patient data are collected during the planned study visits on site using electronic case report forms (e-CRF) hosted by KKS Marburg. The data are entered directly at the participating sites via a web browser to the e-CRF and are transferred via TLS encryption to the central database.

Access to the e-CRF is only allowed for documented trial personnel using individual user accounts.

In a multistage procedure, the given data will be checked electronically for their plausibility and consistency. Even during data collection, implausible data will be flagged automatically by implemented validation checks. In the next stage, detected inconsistencies and missing or implausible data will be clarified with queries and necessary changes will be carried out.

In order to ensure the anonymity of the patient data, the patient data in the e-CRF are recorded with a patient number consisting of a center number and a consecutive number. An allocation list (Rando-Log) containing the patient number and the identifying data of the patient is only kept in the participating site.

Missing data will be addressed by intention-to-treat (ITT) analysis by single and multiple imputations, weighted estimating equations or model-based strategies. Specific details on the handling of missing data will be given in a statistical analysis plan that will be finalized prior to database lock.

### Safety endpoints

Safety data will be summarized by using descriptive statistical methods. All adverse events (AEs) occurring during the conduct of the clinical trial will be monitored carefully and recorded on electronic case report forms (eCRFs). The causality assessment is performed by the investigator of the trial site concerned. In their clinical routine, the participating sites work with standardized questionnaires to systematically elicit information about potential adverse events. All adverse events (AE) are collected in the e-CRF and marked if serious or not. AEs which are frequent and of clinical interest (e.g., hematological and renal toxicities) will be reported in the final publication of the trial results.

All serious adverse events (SAEs) will be reported by the investigators to the Philipps-University of Marburg (KKS Marburg) within 24 h of becoming known. SAEs are recorded in the study-specific safety database. The SAE assessment is performed by KKS Marburg. The collection and handling of serious adverse events (SAE) follow procedures described in the study-specific SAE manual. SAEs are collected in a pharmacovigilance software which is validated for reporting obligations to the authorities.

All suspected adverse reactions related to an investigational medicinal product that are both unexpected and serious (SUSARs) will be notified by KKS Marburg to the competent authority, the ethics committee, and all investigators involved.

The study conduct and safety of participants will be monitored by a *Data and Safety Monitoring Committee (*DSMC). The *DSMC* consists of two clinical experts and one statistician independent from the coordinating investigator supervising the conduct of the study. Furthermore, clinical monitoring of patient data will be performed by on-site or remote by sponsor staff. Sponsor staff specializing in quality management will audit trial sites’ adherence to planned processes on site.

The DSMC will meet via video conference on a regular basis (first meeting 6 months after inclusion of the first patient and once per year after that) and will be responsible for the independent evaluation of the safety of patients taking part in the clinical trial. The DMSC will also check the integrity and the validity of the data and will make recommendations.

The trial steering committee consists of the clinical coordinators for each country (JG, PJ), the sponsor representative (CSB), and the trial statistician (AR).

All participants will be offered continuous follow-up after completion of the trial in the specialized lung transplant clinics in participating centers and are taken into account by patients in the vast majority. In Europe, lifelong center-based follow-up is the standard of care after lung transplantation to assure optimal patient management, quality management, and handling of post-trial complications. In both centers in case or irreversible graft loss, the possibility of re-do transplantation can be offered.

### Dissemination

The results of the trial are planned to be presented at congresses and to be published in a medical journal. Furthermore, trial results are planned to be distributed via newsletter of the German Center of Lung Research. Guidelines of authorship of the Deutsche Forschungsgemeinschaft will be followed. Authorship will be determined according to the number of randomized centers between principal investigators. Trial data will be made available for publicity starting 1 year and ending 10 years after the publication of results upon request to the corresponding author.

There is no plan for using professional writing in the distribution of the results.

## Discussion

This is the first prospectively randomized trial to investigate tailoring of immunosuppression by a DNA virus (torque teno virus) in comparison with conventional therapeutic drug monitoring in lung transplantation.

Viral infection may cause symptoms, but some viruses can be found in healthy individuals without causing disease. Torque teno virus (TTV) is a human DNA virus resulting in asymptomatic viremia. TTV viremia is frequently detected in the general population without associated symptoms or disease. Epstein-Barr virus (EBV) represents another DNA virus, has a high prevalence in adults, and causes latent infection in memory B cells after primary infection, and viremia is usually asymptomatic in the transplant population. The levels of EBV in the blood are correlated with the intensity of immunosuppression. Both TTV and EBV have therefore been suggested as surrogate markers of the net state of immunosuppression. In previous studies, TTV DNA in the blood but not EBV viremia is correlated positively with the intensity of immunosuppression after transplantation. Another argument against the use of EBV load as a surrogate marker of immunosuppression is the influence of valganciclovir on EBV load, a drug commonly used for preventing CMV infection after lung transplantation. Its use may reduce EBV load. In conclusion, TTV but not EBV DNA load better reflects the function of the immune system after lung transplantation. Immunosuppression depends on the type, combinations, and dosing of immunosuppressive treatment.

In lung transplant recipients, a large benefit may be expected by tailoring immunosuppression by DNA virus monitoring because immunosuppression is usually more intense in comparison with other forms of solid organ transplantation. This hypothesis is optimally being studied in a prospective manner in comparison with conventional therapeutic drug monitoring.

The guidance of immunosuppression by monitoring of TTV load will be chosen because in contrast to EBV, and cytomegalovirus (CMV) is expected to be measurable in the blood in the vast majority of lung transplant recipients and monitoring of this virus is therefore suitable for the guidance of immunosuppression. Since less than 25% of recipients are expected to develop CMV viremia during the first postoperative year, CMV load will not be suitable as a monitoring tool for immunosuppression. Furthermore, several publications have demonstrated changes in TTV load in relation to the intensity of immunosuppression [3, 7]. CMV load will be monitored routinely in addition to all recipients, and prophylaxis will be given. However, randomization will be stratified by high-risk CMV status (D+R−) (yes/no) as in the Vienna cohort [4] a lower TTV load was observed during CMV infections. Blinding of patients and/or staff will not be applied as the primary endpoint will be determined by using laboratory values in the CKD-EPI formula.

To compare the results regarding TTV levels between different transplantation centers, it is vital to reliably quantify the concentrations of TTV DNA in a standardized manner. In the VIGILung trial, this will be performed by central analysis of TTV load in a reference lab because real-time PCR is prone to inter-laboratory differences.

The effect size assumed for the sample size calculation was derived from the observed glomerular filtration rate (GFR) changes within 12 months in two recent immunosuppressive trials including reduced calcineurin inhibitor strategies [8, 13]. All endpoints will be assessed by pre-defined definitions [9, 14–18]. The CKD-EPI method was chosen to estimate GFR because it is the most robust creatinine-based method and is established in clinical routine and trials in lung transplantation [19]. CLAD will be diagnosed by a persistent (at least 3 months) decline of FEV1 to 80% of baseline or below after adequate treatment of secondary causes such as infection, acute cellular/antibody-mediated rejection, or airway stenosis.

The authors recognize that the COVID-19 public health emergency may impact the conduct of the trial, e.g., by site personnel or trial participants become infected with COVID-19, quarantines, site closures, and travel limitations. Safety of trial participants is ensured by the prescription of immunosuppressants by local physicians and remote monitoring of patients by telephone and video consultation [20]. During the first wave of the pandemic, shortage in immunosuppressive medications was not noted in Germany and Austria. In-person visits by indications were still possible in both participating centers. Changes in the study visit schedules, missed visits, or patient discontinuations may lead to missing information. Temporary stopping of recruitment may limit the amount of missing data.

The results of this trial might have a large impact on therapeutic strategies for patients after lung transplantation. Furthermore, this study will contribute to improve evidence-based therapy in these patients. Study results are transferable to all other solid organ transplantations.

### Trial status

The trial is recruiting patients since 28 July 2020 according to the VIGILung protocol version V03 from 05 November 2019. Recruitment time is planned to be 39 months.

## Supplementary Information

**Additional file 1.** SPIRIT checklist. Filled out SPIRIT checklist for the VGILung trial protocol.

**Additional file 2.** Translation of the funding information

**Additional file 3.** Translation of the Ethics committee’s vote of Hannover Medical School

**Additional file 4.** Translation of the Ethics committee’s vote of Medical University of Vienna

**Additional file 5.** Tabular view from registration at clinicaltrials.gov concerning WHO trial dataset

## Data Availability

As the study is ongoing, data are not yet available. Materials about the study are available under the registration number: EudraCT-Number 2019-001770-29.

## Abbreviations

AE: Adverse event
AR: Adverse reaction
ATG: Antithymocyte globulin
BAL: Bronchoalveolar lavage
CA: Competent authority
CARV: Community-acquired respiratory viral infections
CKD-EPI: Chronic Kidney Disease Epidemiology Collaboration
CMV: Cytomegalovirus
CNI: Calcineurin inhibitor
CRF: Case report form
CsA: Ciclosporin A
DNA: Deoxyribonucleic acid
DSA: Donor-specific a
DSMC: Data Safety Monitoring Committee
DSUR: Development Safety Update Report
EBV: Epstein-Barr virus
EC: Ethics committee
EC-MPS: Enteric-coated mycophenolate sodium
FEV1: Forced expiratory volume in 1 s
GCP: Good Clinical Practice
GFR: Glomerular filtration rate
HBsAG: Hepatitis B surface antigen
HCV: Hepatitis C virus
HHV-6: Human herpesvirus 6
HIV: Human immunodeficiency virus
HLA: Human leukocyte antigen
ICH: International Conference on Harmonization
IS: Immunosuppression
ITT: Intention-to-treat
LC-MS: Liquid chromatography-mass spectrometry
LTx: Lung transplantation
MMF: Mycophenolate mofetil
MPA: Mycophenolic acid
pO_2_: Oxygen partial pressure
PP: Per-protocol
SAE: Serious adverse event
SaO_2_: Blood-oxygen saturation
SAR: Serious adverse reaction
SmPC: Summary of product characteristics
SpO_2_: SaO_2_ measurement determined by pulse oximetry
SUSAR: Suspected unexpected serious adverse reaction
TAC: Tacrolimus
TBB: Transbronchial biopsy
TDM: Therapeutic drug monitoring
TLC: Total lung capacity
TTV: Torque teno virus
UAR: Unexpected adverse reaction
6MWT: 6-minute walk test
