# Supplementary material for: Viral load-guided immunosuppression after lung transplantation (VIGILung)—study protocol for a randomized controlled trial
Source: Trials. 2021 Jan 11;22:48. doi: 10.1186/s13063-020-04985-w (PMC7798016; doi:10.1186/s13063-020-04985-w)
Supplement: Supplementary file 1 — Additional file 1. SPIRIT checklist. Filled out SPIRIT checklist for the VGILung trial protocol. [file 13063_2020_4985_MOESM1_ESM.zip › Appendix to SPIRIT-checklist_Patinfo andConsentR1 (add1).pdf]

Prüfstelle: Klinik für Pneumologie
Medizinische Hochschule Hannover
Carl-Neuberg-Str. 1
30625 Hannover
Tel. 0511-532-4681 (Koordinator)

Prüfarzt: ______________________________ **Tel.:**______________________

EUDRACT-Nr.: 2019-001770-29
Prüfplancode: KKS-256

Leiter der klinischen Prüfung Deutschland:
Prof. Dr. med. Jens Gottlieb
Klinik für Pneumologie
Medizinische Hochschule Hannover
Carl-Neuberg-Str. 1
30625 Hannover

Leiter der klinischen Prüfung Österreich:
PD Dr. med. Peter Jaksch
Klinische Abteilung für Thoraxchirurgie
Medizinische Universität Wien
Währinger Gürtel 18-20
1090 Wien

Viruslast gesteuerte Immunsuppression nach Lungentransplantation (VIGILung)

Eine offene, randomisierte, kontrollierte, parallel-gruppen, multizentrische Studie

Patienteninformation

Sehr geehrte Patientin, sehr geehrter Patient,

Sie sind erfolgreich lungentransplantiert und befinden sich in aktiver Nachsorge in Ihrem Transplantationszentrum. Wie Sie wissen, ist eine Unterdrückung der körpereigenen Abwehr mit Medikamenten (sogenannte Immunsuppression) notwendig, damit ihr Spenderorgan nicht abgestoßen wird. Ihr Arzt stellt Ihnen heute eine klinische Studie vor, in der die Steuerung dieser Immunsuppression mit einem neuen Bluttest untersucht werden soll.
Wir möchten Sie fragen, ob Sie bereit sind an der nachfolgend beschriebenen klinischen Prüfung, der sogenannten VIGILung-Studie, teilzunehmen. Sie erhalten im Folgenden Informationen zum Ablauf dieser klinischen Prüfung (Studie).
Bevor Sie entscheiden, ob Sie an der Studie teilnehmen möchten, ist es wichtig, dass Sie verstehen, warum die Studie durchgeführt wird und was sie mit sich bringt. Bitte nehmen Sie sich Zeit, die folgenden Informationen aufmerksam zu lesen. Sie können jederzeit den Studienarzt ansprechen, falls Ihnen etwas unklar ist oder falls Sie weitere Informationen wünschen.
Klinische Forschung ist notwendig, um die Behandlung von Krankheiten stetig zu verbessern. Sie darf nur unter Beachtung strenger nationaler und internationaler Gesetze und Richtlinien erfolgen. Klinische Studien sind eine Voraussetzung dafür, dass wirksamere und risikoärmere Behandlungsverfahren erforscht und entwickelt werden können.
Die VIGILung Studie wird an mindestens 3 Zentren in Deutschland und Österreich durchgeführt; es sollen insgesamt 144 Personen daran teilnehmen. Die Studie wurde von erfahrenen Transplantations-Spezialisten und fachkundigen Ärzten aus diesen Zentren geplant. Die Studie wird gemeinsam von der Medizinischen Hochschule Hannover, Klinik für Pneumologie und der Philipps-Universität Marburg/KKS Marburg durchgeführt und finanziert durch die Deutsche Forschungsgemeinschaft (DFG).

Ihre Teilnahme an dieser klinischen Studie ist freiwillig. Sie werden in diese Studie also nur dann einbezogen, wenn Sie dazu schriftlich Ihre Einwilligung erklären. Sofern Sie nicht an der

klinischen Studie teilnehmen oder später aus ihr ausscheiden möchten, entstehen Ihnen daraus keine Nachteile.

Der nachfolgende Text soll Ihnen die Ziele und den Ablauf erläutern. Anschließend wird ein Prüfarzt das Aufklärungsgespräch mit Ihnen führen. Bitte zögern Sie nicht, alle Punkte anzusprechen, die Ihnen unklar sind. Sie werden danach ausreichend Bedenkzeit erhalten, um über Ihre Teilnahme zu entscheiden.

1. Warum wird diese Studie durchgeführt?

Diese Studie dient zur Gewinnung von Informationen über eine Steuerung der Immunsuppression nach Lungentransplantation durch ein Labor Testverfahren.
Sie müssen nach der Transplantation lebenslang Medikamente zur Unterdrückung der körpereigenen Abwehr (Immunsuppressiva) einnehmen, um eine Abstoßung des Lungentransplantats zu vermeiden. Diese Medikamente (z.B. Tacrolimus, Mycophenolat Mofetil (MMF) und Prednisolon) steigern aber auch die Infektanfälligkeit und können Erkrankungen begünstigen bzw. sich auf den Verlauf bereits bestehender Krankheiten negativ auswirken (z.B. Diabetes, hoher Blutdruck, Nierenschädigung).
Um ein optimales Gleichgewicht zwischen Wirkung (vermindertes Abstoßungsrisiko) und Nebenwirkungen (z.B. erhöhte Infektanfälligkeit, Nierenschädigung) zu erreichen, wird üblicherweise der Spiegel der Medikamente im Blut (z.B. von Tacrolimus) bestimmt und die Dosis nach den Ergebnissen der Blutspiegel angepasst. Dies stellt die Standardtherapie in der Steuerung der Immunsuppression dar.
Trotz intensiver Kontrollen der Medikamentenspiegel treten bei etwa 30 bis 40 % der Patienten akute Abstoßungen im ersten Jahr nach der Transplantation auf. Gleichzeitig reduziert sich die Nierenfunktion der Transplantatempfänger innerhalb des ersten Jahres um 30-50 % als Ausdruck einer Nebenwirkung dieser Medikamente. Die Einschränkung der Nierenfunktion ist abhängig von der Höhe der Immunsuppression (Medikamente gegen Abstoßung). In der klinischen Forschung wird seit Jahren versucht Tests zu entwickeln, die über die Medikamentenspiegel hinaus etwas über die Wirksamkeit der Immunsuppression im Patienten aussagen. Wenn solche Tests vorliegen, könnte damit die Immunsuppression bei gleicher Wirksamkeit weniger unerwünschte Wirkungen erzielen.
Die Bestimmung des Torque-Teno-Virus (TTV) im Vollblut scheint ein geeigneter Labortest dafür zu sein. Bei fast allen Transplantierten werden diese Viren im Blut gefunden. Wichtig ist, dass diese Viren zu keiner eigenständigen Krankheit des Transplantatempfängers führen. Die Anzahl der Viren im Blut ist abhängig von der Intensität der Immunsuppression. Die Zahl dieser Viren im Blut wurde deshalb in einigen Zentren als Zielwert für das Maß der Immunsuppression genutzt. In dieser Studie soll die Sicherheit und Wirksamkeit einer Immunsuppression auf Basis der zusätzlichen Überwachung der Immunsuppression mit Bestimmung der Torque-Teno-Viren im Blut im Vergleich zur Standardüberwachung (d.h. Kontrolle der Medikamentenspiegel im Blut) untersucht werden.
Der Endpunkt der Studie ist die Messung der Nierenfunktion, deren Messung durch Blutwerte die beiden Behandlungsgruppen nach einem Jahr miteinander vergleicht.

2. Erhalte ich die personalisierte Steuerung der Immunsuppression auf jeden Fall?

Man wird Sie nach dem Zufallsprinzip (wie beim Werfen einer Münze oder beim Würfeln) entweder der Steuerung der Immunsuppression nach dem Standard (d.h. Medikamentenspiegel im Blut) oder einer zusätzlichen Steuerung der Immunsuppression über die TTV-Viruslast zuteilen. Dieses Verfahren wird „Randomisierung“ genannt, 50% der Patienten erhalten weiterhin die Standardtherapie und bei 50% der Patienten wird nach der Randomisierung die TTV-Last bestimmt und der Tacrolimus-Zielspiegel ggf. zusätzlich angepasst

In der Studie werden also 2 Gruppen verglichen, die aber mindestens 12 Monate behandelt werden:

Gruppe 1: Steuerung der Tacrolimus-Zielspiegel im Blut und zusätzlicher Bestimmung der Zahl an TTV-Viren im Blut (sog. TTV-Last)
Gruppe 2: Standardtherapie mit Steuerung der Tacrolimus-Zielspiegel im Blut

In beiden Gruppen wird die Tacrolimus-Dosis entsprechend angepasst an die Ergebnisse der regelmäßigen Bestimmung der Talspiegel.
In der Gruppe 1 mit zusätzlicher Bestimmung der TTV-Last wird nach vorbestimmten Regeln die Höhe der Zielspiegel zusätzlich angepasst.

3. Wie ist der Ablauf der Studie und was muss ich bei Teilnahme beachten?

Der Ablauf der Studie mit der Planung der Studienbesuche (Visiten) richtet sich nach der Routine in der Nachsorge nach Lungentransplantation, wie sie in den teilnehmenden Zentren üblich ist.

Studienbeginn, Visite 1 (ca. 1 Monat nach Transplantation)

Sobald Sie die Einwilligungserklärung unterschrieben haben, werden Sie einer Reihe von Untersuchungen unterzogen, die routinemäßig für Ihre Erkrankung durchgeführt werden:

- Ihre Krankengeschichte sowie Ihr derzeitiger Krankheitszustand wird überprüft.
- Alle Medikamente, die Sie zurzeit anwenden, werden überprüft.
- Eine körperliche Untersuchung findet statt (einschl. Körpergewicht und Größe).
- Ihre sog. „Vitalzeichen“ werden untersucht (Blutdruck, Herzfrequenz, Körpertemperatur).
- Man wird Ihnen Blutproben von wenigen Millilitern (ca. 12.5 ml) für Labortests zur Überprüfung der Anzahl Ihrer Blutkörperchen, der Nieren- und Leberfunktion, der Anzeichen für eine Infektion, Blutfette, Tacrolimus Talspiegel, TTV-Last, etc. entnehmen.
- Lungenfunktionstests
    - Spirometrie -wieviel Luft Sie ein- und ausatmen können (Vitalkapazität und Ein-Sekundenkapazität)
- 6-Minuten-Gehtest (6MWT) - es wird getestet, wie weit Sie in 6 Minuten laufen können
- Bronchoskopie mit Bronchialspülung (BAL) und transbronchialer Biopsie (TBB)
- Fragebogen zu Ihrer Gesundheit
- Wenn Sie eine Frau im gebärfähigen Alter sind, wird vor Ihrer Aufnahme in die Studie ein Schwangerschaftstest durchgeführt.

Ihr Arzt bittet Sie zudem, einen zweiseitigen Fragebogen zur Beurteilung Ihrer gesundheitsbezogenen Lebensqualität auszufüllen.

Die TTV-Last wird in beiden Gruppen bis zur Randomisierung (Einteilung nach Zufallsprinzip) bestimmt, da auch bei den Patienten in der Standardtherapie Gruppe sichergestellt sein muss, dass der TT-Virus bei Ihnen nachgewiesen werden kann. Die eigentliche Randomisierung findet 3 Monate nach Transplantation statt, nachdem bekannt ist, wie hoch ihre TTV-Last ist.

Visite 2 (2 Monate nach Transplantation)

- Eine körperliche Untersuchung findet statt (einschl. Körpergewicht).
- Ihre Vitalzeichen werden untersucht (Blutdruck, Herzfrequenz, Körpertemperatur).
- Man wird Ihnen Blutproben von wenigen Millilitern (ca. 12.5 ml) für Labortests zur Überprüfung der Anzahl Ihrer Blutkörperchen, der Nieren- und Leberfunktion, der Anzeichen für eine Infektion, Blutfette, Tacrolimus Talspiegel, TTV-Last, etc. entnehmen.
- Lungenfunktionstests
    - Spirometrie -wieviel Luft Sie ein- und ausatmen können (Vital- und Ein-Sekundenkapazität)
- Fragebogen zu Ihrer Gesundheit

Visite 3 (3 Monate nach Transplantation)

- Eine körperliche Untersuchung findet statt (einschl. Körpergewicht).
- Ihre Vitalzeichen werden untersucht (Blutdruck, Herzfrequenz, Körpertemperatur).
- Man wird Ihnen Blutproben von wenigen Millilitern (ca. 12.5 ml) für Labortests zur Überprüfung der Anzahl Ihrer Blutkörperchen, der Nieren- und Leberfunktion, der Anzeichen für eine Infektion, Blutfette, Tacrolimus Talspiegel, TTV-Last, etc. entnehmen.
- Lungenfunktionstests
  - Spirometrie -wieviel Luft Sie ein- und ausatmen können (Vital- und Ein-Sekundenkapazität)
- 6-Minuten-Gehtest (6MWT) - es wird getestet, wie weit Sie in 6 Minuten laufen können
- Bronchoskopie mit Bronchialspülung (BAL) und transbronchialer Biopsie (TBB)
- Fragebogen zu Ihrer Gesundheit

Visiten 4-6 (3,6 und 9 Monate nach Randomisierung)

Sie haben jeweils nach dem dritten, sechsten und neunten Monat nach Randomisierung Nachbeobachtungsbesuche bei Ihrem Arzt. Bei jedem Besuchstermin wird eine Reihe von Routinetests durchgeführt:

- Eine körperliche Untersuchung findet statt (einschl. Körpergewicht).
- Ihre Vitalzeichen werden untersucht (Blutdruck, Herzfrequenz, Körpertemperatur).
- Lungenfunktionstests (Spirometrie)
- Bluttests (Anzahl der Blutkörperchen, Nieren- und Leberfunktion, Anzeichen für eine Infektion, etc.)
- Bronchoskopie mit Bronchialspülung (BAL) und transbronchialer Biopsie (TBB)
- Fragebogen zu Ihrer Gesundheit

Visite 7 (Abschluss 12 Monate nach Randomisierung)

Dies ist der letzte Studienbesuch.
Sie werden erneut gebeten, den Fragebogen zu Ihrer Gesundheit auszufüllen und werden einer Reihe von Routinetests unterzogen:

- Eine körperliche Untersuchung findet statt (einschl. Körpergewicht).
- Ihre Vitalzeichen werden untersucht (Blutdruck, Herzfrequenz, Körpertemperatur).
- Lungenfunktionstests
  - Spirometrie
- Bronchoskopie mit Bronchialspülung (BAL) und transbronchialer Biopsie (TBB)
- 6-Minuten-Gehtest - es wird getestet, wie weit Sie in 6 Minuten laufen können,
- Bluttests zur Überprüfung der Anzahl der Blutkörperchen, der Nieren- und Leberfunktion und der Anzeichen für eine Infektion
- Fragebogen zu Ihrer Gesundheit

4. Welchen persönlichen Nutzen habe ich von der Teilnahme an der Studie?

Wenn Sie der Gruppe zugeteilt werden, die die TTV-Last gesteuerte Immunsupression erhält, kann es sein, dass Sie einen direkten medizinischen Nutzen aus der Teilnahme an dieser Studie ziehen, dies muss aber nicht der Fall sein. Man hofft zwar, dass die neue Steuerung der Immunsupression zu weniger unerwünschten Wirkungen und Infekten führt, aber es kann auch sein, dass Sie durch Ihre Teilnahme nicht den erhofften Nutzen haben werden.
Wenn Sie der Standard-Gruppe zugeteilt werden, werden Sie die beste verfügbare Behandlung nach dem Versorgungsstandard erhalten, und Ihr gesundheitlicher Zustand wird während der häufigen Studientermine engmaschig überwacht. Ihr Studienarzt kann Änderungen bei Ihren üblichen Medikamenten vornehmen, um Änderungen Ihres Gesundheitszustands zu behandeln.

5. Welche Risiken sind mit der Teilnahme an der Studie verbunden?

Mit der Teilnahme an der Studie sind keine zusätzlich zu der üblichen klinischen Versorgung von Patienten mit Zustand nach Lungentransplantation auftretenden Risiken verbunden.

Bitte teilen Sie den Mitarbeitern der Prüfstelle *alle* Beschwerden, Erkrankungen oder Verletzungen mit, die im Verlauf der klinischen Prüfung (Studie) auftreten. Falls diese schwerwiegend sind, teilen Sie den Mitarbeitern der Prüfstelle diese bitte umgehend mit, ggf. telefonisch.
Mögliche Belastungen und Risiken durch die Anwendung der immunsuppressiven Medikamente können Sie der Auflistung im Anhang dieser Patienteninformation (Seite 13-21) entnehmen.

6. Welche anderen Behandlungsmöglichkeiten gibt es außerhalb der Studie?

Sollten Sie sich nicht für eine Teilnahme an dieser Studie entscheiden werden Sie die medizinische Standardversorgung erhalten.

7. Wer darf an dieser klinischen Studie nicht teilnehmen?

An dieser klinischen Studie dürfen Sie nicht teilnehmen, wenn Sie gleichzeitig an anderen klinischen Studien oder anderen klinischen Forschungsprojekten teilnehmen.

Weibliche Teilnehmer

Wenn Sie schwanger sind oder stillen, dürfen Sie an dieser Studie nicht teilnehmen.

Zu Beginn der klinischen Studie müssen sich deshalb alle Frauen einem Schwangerschaftstest unterziehen. Davon ausgenommen sind Frauen nach den Wechseljahren oder solche, die operativ sterilisiert wurden. Durch einen Schwangerschaftstest kann jedoch eine Schwangerschaft erst einige Tage nach der Empfängnis verlässlich nachgewiesen werden.
Im Falle Ihrer Teilnahme an dieser klinischen Studie müssen Sie zuverlässige Maßnahmen zur Schwangerschaftsverhütung anwenden. Sie müssen zwei mit Ihrem Hausarzt besprochene Verhütungsmethoden anwenden, wie z.B. orale hormonelle Antikontrazeptiva („die Pille“), doppelte Verhütungsmethode (Kondom und Diaphragma mit einer vaginal spermiziden Substanz (Creme, Gel, Zäpfchen), Injektionspräparate (Depot-Spritzen), um eine Schwangerschaft während der Studie zu verhindern.
Sollten Sie während der klinischen Studie schwanger werden oder den Verdacht haben, dass Sie schwanger geworden sind, müssen Sie umgehend den Prüfarzt informieren.

8. Entstehen für mich Kosten durch die Teilnahme an der klinischen Studie? Erhalte ich eine Aufwandsentschädigung?

Durch Ihre Teilnahme an dieser klinischen Studie entstehen für Sie keine zusätzlichen Kosten.

9. Bin ich während der klinischen Studie versichert?

Während der klinischen Prüfung (Studie) sind Sie versichert.
Der Umfang des Versicherungsschutzes ergibt sich aus den Versicherungsunterlagen, die Sie ausgehändigt bekommen.
Wenn Sie vermuten, dass durch die Teilnahme an der klinischen Studie Ihre Gesundheit geschädigt oder bestehende Leiden verstärkt wurden, müssen Sie dies unverzüglich dem Versicherer

Name und Anschrift der Versicherung:
HDI Global SE
Niederlassung Düsseldorf
Am Schönenkamp 45
40599 Düsseldorf
Telefon: 0211 - 74825404
Fax: 0211 - 7482465
Versicherungsnummer: 57 010312 03019

direkt anzeigen, gegebenenfalls mit Unterstützung durch Ihren Prüfarzt, um Ihren Versicherungsschutz nicht zu gefährden. Sofern Ihr Prüfarzt Sie dabei unterstützt, erhalten Sie eine Kopie der Meldung. Sofern Sie Ihre Anzeige direkt an den Versicherer richten, informieren Sie bitte zusätzlich Ihren Prüfarzt.

Bei der Aufklärung der Ursache oder des Umfangs eines Schadens müssen Sie mitwirken und alles unternehmen, um den Schaden abzuwenden und zu mindern.

Während der Dauer der klinischen Studie dürfen Sie sich einer anderen medizinischen Behandlung – außer in Notfällen – nur nach vorheriger Rücksprache mit dem Prüfarzt unterziehen. Von einer erfolgten Notfallbehandlung müssen Sie den Prüfarzt unverzüglich unterrichten.

Sie erhalten ein Exemplar der Versicherungsbedingungen.

Wir weisen ferner darauf hin, dass Sie auf dem Weg von und zur Prüfstelle in folgender Weise versichert sind.

Name und Anschrift der Versicherung:
SV SparkassenVersicherung AG
Postfach 3120
65021 Wiesbaden
Telefon: 0611 - 178-100
Fax: 0611 - 178-109
Versicherungsnummer: 50078024001

10. Werden mir neue Erkenntnisse während der klinischen Studie mitgeteilt?

Sie werden über neue Erkenntnisse, die in Bezug auf diese klinische Studie bekannt werden und die für Ihre Bereitschaft zur weiteren Teilnahme wesentlich sein können, informiert. Auf dieser Basis können Sie dann Ihre Entscheidung zur weiteren Teilnahme an dieser klinischen Studie überdenken.

11. Kann meine Teilnahme an der klinischen Studie vorzeitig beendet werden?

Sie können jederzeit, auch ohne Angabe von Gründen, Ihre Teilnahme beenden, ohne dass Ihnen dadurch irgendwelche Nachteile entstehen.

Unter gewissen Umständen ist es aber auch möglich, dass der Prüfarzt oder die Studienleitung entscheiden Ihre Teilnahme an der klinischen Studie vorzeitig zu beenden, ohne dass Sie auf die Entscheidung Einfluss haben. Die Gründe hierfür können z. B. sein:

- Ihre weitere Teilnahme an der klinischen Studie ist ärztlich nicht mehr vertretbar;
- es wird die gesamte klinische Studie abgebrochen.

Sofern Sie sich dazu entschließen, vorzeitig aus der klinischen Studie auszuscheiden, oder Ihre Teilnahme aus einem anderen der genannten Gründe vorzeitig beendet wird, ist es für Ihre eigene Sicherheit wichtig, dass Sie sich einer empfohlenen abschließenden Kontrolluntersuchung unterziehen.

Diese Untersuchung wird standardmäßige Lungenfunktionstests (Spirometrie, Ganzkörper-Plethysmographie („Lungenfunktionskabine") und Diffusionskapazität), eine Röntgen-aufnahme des Brustkorbs, Bronchienspiegelungen, einen 6-Minuten-Gehtest und Blutproben umfassen. Dies sind alles Routineuntersuchungen nach einer Lungentransplantation. Ihr Arzt wird Sie zudem bitten, den Studienfragebogen zu Ihrer Gesundheit auszufüllen.

12. Was geschieht mit meinen Daten?

Während der klinischen Studie werden medizinische Befunde und persönliche Informationen von Ihnen erhoben und in der Prüfstelle in Ihrer persönlichen Akte niedergeschrieben oder elektronisch gespeichert. Die für die klinische Studie wichtigen Daten werden zusätzlich in „pseudonymisierter" Form gespeichert, ausgewertet und gegebenenfalls weitergegeben.

Pseudonymisiert bedeutet, dass keine Angaben von Namen oder Initialen verwendet werden, sondern nur ein Nummern- und/oder Buchstabencode.

Die Daten sind gegen unbefugten Zugriff gesichert. Eine Entschlüsselung erfolgt nur unter den vom Gesetz vorgeschriebenen Voraussetzungen.

Das Arzneimittelgesetz enthält nähere Vorgaben für den erforderlichen Umfang der Einwilligung in die Datenerhebung und -verwendung. Einzelheiten entnehmen Sie bitte der Einwilligungserklärung, die im Anschluss an diese Patienteninformation abgedruckt ist.

13. Was geschieht mit meinen Blutproben zur Bestimmung der TTV-Last / weitere biologische Proben?

Die Blutproben zur Bestimmung der TTV-Last, Proben der bronchialen Spülflüssigkeit und Gewebeproben werden ebenso wie die Daten in pseudonymisierter Form, also ohne Angaben von Namen oder Initialen aufbewahrt.

Blutproben zur Bestimmung der TTV-Viruslast werden ausschließlich für diese klinische Prüfung verwendet. Dafür werden Blutproben (600 µl) nach Wien (Prof. Elisabeth Puchhammer, Medizinische Universität Wien, Zentrum für Virologie, Probenannahme Zi. 301, Kinderspitalgasse 15, A-1090 Wien) versendet und dort ausgewertet.
Die Aufbewahrung eventueller Restmengen erfolgt bis zum Abschluss der klinischen Prüfung.

Die Aufbewahrung weiterer biologischer Proben erfolgt lokal in Ihrem Transplantations-zentrum - für die MHH: Hannover Unified Biobank (HUB).
Serumproben, Urinproben, Gewebeproben und Proben der bronchialen Spülflüssigkeit werden für diese klinische Prüfung, möglicherweise aber auch für weitere Fragestellungen verwendet. Mit solchen weitergehenden Untersuchungen sollen Fragen geklärt werden, die sich noch zu einem späteren Zeitpunkt stellen können.

14. An wen wende ich mich bei weiteren Fragen?

Beratungsgespräche an der Prüfstelle

Sie haben stets die Gelegenheit zu weiteren Beratungsgesprächen mit dem auf Seite 1 genannten oder einem anderen Prüfarzt.

Kontaktstelle

Es existiert außerdem eine Kontaktstelle bei der zuständigen Bundesoberbehörde. Teilnehmer an klinischen Prüfungen, ihre gesetzlichen Vertreter oder Bevollmächtigten können sich an diese Kontaktstelle wenden:

Bundesinstitut für Arzneimittel und Medizinprodukte
Fachgebiet Klinische Prüfung / Inspektionen
Kurt-Georg-Kiesinger-Allee 3
53175 Bonn

Telefon: 0228 / 207-4318 Fax: 0228 / 207-4355
e-mail: klinpruefung@bfarm.de

Prüfstelle: Klinik für Pneumologie
Medizinische Hochschule Hannover
Carl-Neuberg-Str. 1
30625 Hannover
Tel. 0511-532-4681 (Koordinator)

Prüfarzt: ________________________________ **Tel.:**________________________

EUDRACT-Nr.: 2019-001770-29
Prüfplancode: KKS-256

Leiter der klinischen Prüfung Deutschland:
Prof. Dr. med. Jens Gottlieb
Klinik für Pneumologie
Medizinische Hochschule Hannover
Carl-Neuberg-Str. 1
30625 Hannover

Leiter der klinischen Prüfung Österreich:
PD Dr. med. Peter Jaksch
Klinische Abteilung für Thoraxchirurgie
Medizinische Universität Wien
Währinger Gürtel 18-20
1090 Wien

Viruslast gesteuerte Immunsuppression nach Lungentransplantation (VIGILung)

Eine offene, randomisierte, kontrollierte, parallel-gruppen, multizentrische Studie

Einwilligungserklärung

....
.
Name des Patienten in Druckbuchstaben

geb. am .... Teilnehmer-Nr. ....

Ich bin in einem persönlichen Gespräch durch den Prüfarzt

....
.
Name der Ärztin/des Arztes

ausführlich und verständlich über die geplante Behandlung sowie über Wesen, Bedeutung, Risiken und Tragweite der klinischen Prüfung aufgeklärt worden. Ich habe darüber hinaus den Text der Patienteninformation sowie die hier nachfolgend abgedruckte Datenschutzerklärung gelesen und verstanden. Ich hatte die Gelegenheit, mit dem Prüfarzt über die Durchführung der klinischen Prüfung zu sprechen. Alle meine Fragen wurden zufrieden stellend beantwortet.

Möglichkeit zur Dokumentation zusätzlicher Fragen seitens des Patienten oder sonstiger Aspekte des Aufklärungsgesprächs:

Ich hatte ausreichend Zeit, mich zu entscheiden.

Mir ist bekannt, dass ich jederzeit und ohne Angabe von Gründen meine Einwilligung zur Teilnahme an der Prüfung zurückziehen kann (mündlich oder schriftlich), ohne dass mir daraus Nachteile entstehen.

Datenschutz:

Mir ist bekannt, dass bei dieser klinischen Prüfung personenbezogene Daten, insbesondere medizinische Befunde über mich erhoben, gespeichert und ausgewertet werden sollen. Die Verwendung der Daten erfolgt nach gesetzlichen Bestimmungen und setzt vor der Teilnahme an der klinischen Prüfung folgende freiwillig abgegebene Einwilligungserklärung voraus, das heißt ohne die nachfolgende Einwilligung kann ich nicht an der klinischen Prüfung teilnehmen.

1. Ich erkläre mich damit einverstanden, dass im Rahmen dieser klinischen Prüfung personenbezogene Daten, insbesondere Angaben über meine Gesundheit und meine ethnische Herkunft, über mich erhoben und in Papierform sowie auf elektronischen Datenträgern bei/in der auf Seite 1 dieser Patienteninformation und Einwilligungserklärung genannten Prüfstelle aufgezeichnet werden. Soweit erforderlich, dürfen die erhobenen Daten pseudonymisiert (verschlüsselt) weitergegeben werden:
   a) an die Philipps-Universität Marburg, KKS oder eine von diesem beauftragte Stelle zum Zwecke der wissenschaftlichen Auswertung,
   b) ggf. an nationale und internationale Zulassungsbehörden,
   c) im Falle unerwünschter Ereignisse: an die Philipps-Universität Marburg, KKS an die jeweils zuständige Ethik-Kommission und die zuständige Bundesoberbehörde (in Deutschland: Bundesinstitut für Arzneimittel und Medizinprodukte, in Österreich: Bundesamt für Sicherheit im Gesundheitswesen), sowie von diesen an die Europäische Datenbank.

2. Außerdem erkläre ich mich damit einverstanden, dass autorisierte und zur Verschwiegenheit verpflichtete Beauftragte des Sponsors sowie die zuständigen in- und ausländischen Überwachungsbehörden in meine beim Prüfarzt vorhandenen personenbezogenen Daten, insbesondere meine Gesundheitsdaten, Einsicht nehmen, soweit dies für die Überprüfung der ordnungsgemäßen Durchführung der Studie notwendig ist. Für diese Maßnahme entbinde ich den Prüfarzt von der ärztlichen Schweigepflicht.

3. Ich bin darüber aufgeklärt worden, dass ich jederzeit die Teilnahme an der klinischen Prüfung beenden kann. Die Einwilligung zur Erhebung und Verarbeitung meiner personenbezogenen Daten, insbesondere der Angaben über meine Gesundheit, ist jedoch unwiderruflich. Ich weiß, dass im Falle eines Widerrufs zur Teilnahme an der klinischen Prüfung die bis zu diesem Zeitpunkt gespeicherten Daten weiterhin verwendet werden dürfen, soweit dies erforderlich ist, um
   a) Wirkungen des zu prüfenden Arzneimittels festzustellen,
   b) sicherzustellen, dass meine schutzwürdigen Interessen nicht beeinträchtigt werden,

4. Ich erkläre mich damit einverstanden, dass meine Daten nach Beendigung oder Abbruch der Prüfung mindestens zehn Jahre aufbewahrt werden, wie es die Vorschriften über die klinische Prüfung von Arzneimitteln bestimmen. Danach werden meine personenbezogenen Daten gelöscht, soweit nicht gesetzliche, satzungsmäßige oder vertragliche Aufbewahrungsfristen entgegenstehen.

5. Ich bin über folgende gesetzliche Regelung informiert: Falls ich meine Einwilligung, an der Studie teilzunehmen, widerrufe, müssen alle Stellen, die meine personenbezogenen Daten, insbesondere Gesundheitsdaten, gespeichert haben, unverzüglich prüfen, inwieweit die gespeicherten Daten für die in Nr. 3 a) bis c) genannten Zwecke noch erforderlich sind.

   Nicht mehr benötigte Daten sind unverzüglich zu löschen.

6. Ich bin damit einverstanden, dass Gesundheitsdaten von mitbehandelnden Ärzten / von folgenden Ärzten
   ....
   Name(n)
   erhoben werden, soweit dies für die ordnungsgemäße Durchführung und Überwachung der Studie notwendig ist. Insoweit entbinde ich diese Ärzte von der Schweigepflicht. *(Falls nicht gewünscht, bitte streichen.)*

7. Ich bin damit einverstanden, dass mein Hausarzt

   ....
   Name
   über meine Teilnahme an der klinischen Prüfung informiert wird *(falls nicht gewünscht, bitte streichen)*.

Information für Studienteilnehmer gemäß Europäischer Datenschutz-Grundverordnung (gültig ab 25.05.2018)[1] für medizinische Forschungsvorhaben

Hiermit möchten wir Sie über die in der Datenschutz-Grundverordnung (= DS-GVO) festgelegten Rechte informieren (Artikel 12 ff. DS-GVO):

Rechtsgrundlage
Die Rechtsgrundlage zur Verarbeitung der Sie betreffenden personenbezogenen Daten bilden bei klinischen Studien (einschließlich klinischer Prüfungen) Ihre freiwillige schriftliche Einwilligung gemäß DS-GVO sowie der Deklaration von Helsinki (Erklärung des Weltärztebundes zu den ethischen Grundsätzen für die medizinische Forschung am Menschen) und der Leitlinie für Gute Klinische Praxis. Bei Arzneimittel-Studien ist zusätzlich das Arzneimittelgesetz, bei Medizinprodukte-Studien das Medizinproduktegesetz anzuwenden.

Bezüglich Ihrer Daten haben Sie folgende Rechte (Artikel 13 ff. DS-GVO):

Recht auf Auskunft
Sie haben das Recht auf Auskunft über die Sie betreffenden personenbezogenen Daten, die im Rahmen der klinischen Studie erhoben, verarbeitet oder ggf. an Dritte übermittelt werden (Aushändigen einer *kostenfreien* Kopie) (Artikel 15 DS-GVO).

Recht auf Berichtigung
Sie haben das Recht Sie betreffende unrichtige personenbezogene Daten berichtigen zu lassen (Artikel 16 und 19 DS-GVO).

Recht auf Löschung
Sie haben das Recht auf Löschung Sie betreffender personenbezogener Daten, z.B. wenn diese Daten für den Zweck, für den sie erhoben wurden, nicht mehr notwendig sind oder Sie Ihre Einwilligung widerrufen, auf die sich die Verarbeitung Ihrer Daten stützt. Die Rechtmäßigkeit der aufgrund der Einwilligung bis zum Widerruf erfolgten Verarbeitung wird hiervon nicht berührt." (Artikel 7, 17 und 19 DS-GVO).

Recht auf Einschränkung der Verarbeitung
Unter bestimmten Voraussetzungen haben Sie das Recht, die Einschränkung der Verarbeitung zu verlangen, d.h. die Daten dürfen nur gespeichert, aber nicht verarbeitet werden. Dies müssen Sie beantragen. Wenden Sie sich hierzu bitte an Ihren Prüfer oder an den Datenschutzbeauftragten des Prüfzentrums (Artikel 18 und 19 DS-GVO).

---

[1] Verordnung (EU) 2016/679 des Europäischen Parlaments und des Rates vom 27. April 2016 zum Schutz natürlicher Personen bei der Verarbeitung personenbezogener Daten, zum freien Datenverkehr und zur Aufhebung der Richtlinie 95/46/EG (Datenschutz-Grundverordnung)

Im Falle der Berichtigung, Löschung, Einschränkung der Verarbeitung werden zudem all jene benachrichtigt, die Ihre Daten erhalten haben (Artikel 17 Absatz 2 und Artikel 19 DS-GVO).

Recht auf Datenübertragbarkeit
Sie haben das Recht, die Sie betreffenden personenbezogenen Daten, die Sie dem Verantwortlichen für die klinische Studie bereitgestellt haben, zu erhalten. Damit können Sie beantragen, dass diese Daten entweder Ihnen oder, soweit technisch möglich, einer anderen von Ihnen benannten Stelle übermittelt werden (Artikel 20 DS-GVO).

Widerspruchsrecht
Sie haben das Recht, jederzeit gegen konkrete Entscheidungen oder Maßnahmen zur Verarbeitung der Sie betreffenden personenbezogenen Daten Widerspruch einzulegen (Art 21 DS-GVO). Eine solche Verarbeitung findet anschließend grundsätzlich nicht mehr statt.

Einwilligung zur Verarbeitung personenbezogener Daten und Recht auf Widerruf dieser Einwilligung
Die Verarbeitung Ihrer personenbezogenen Daten ist nur mit Ihrer Einwilligung rechtmäßig (Artikel 6 DS-GVO). Sie haben das Recht, Ihre Einwilligung zur Verarbeitung personenbezogener Daten jederzeit zu widerrufen. Die Rechtmäßigkeit der aufgrund der Einwilligung bis zum Widerruf erfolgten Verarbeitung wird hiervon nicht berührt (Artikel 7 Absatz 3 DS-GVO).

Benachrichtigung bei Verletzung des Schutzes personenbezogener Daten („Datenschutzpannen“)
Hat eine Verletzung des Schutzes personenbezogener Daten voraussichtlich ein hohes Risiko für Ihre persönlichen Rechte und Freiheiten zur Folge, so werden Sie unverzüglich benachrichtigt (Artikel 34 DS-GVO).

Übermittlungen personenbezogener Daten an Drittländer oder an internationale Organisationen
Bei der Übermittlung Ihrer Daten an Länder oder Organisationen außerhalb der EU besteht möglicherweise ein niedrigeres Datenschutzniveau als im Inland (Artikel 44-50 DS-GVO).

Möchten Sie eines dieser Rechte in Anspruch nehmen, wenden Sie sich bitte an Ihren Prüfer oder an den Datenschutzbeauftragten Ihres Prüfzentrums. Außerdem haben Sie das **Recht, Beschwerde bei der/den Aufsichtsbehörde/n einzulegen,** wenn Sie der Ansicht sind, dass die Verarbeitung der Sie betreffenden personenbezogenen Daten gegen die DS-GVO verstößt (**siehe Kontaktdaten**).

KONTAKTDATEN

Datenschutz: Kontaktdaten Prüfzentrum

Datenschutzbeauftragte/r		Datenschutz-Aufsichtsbehörde	
ggf. Name:	Dr. Synia Weber	ggf. Name:	Landesbeauftragte für den Datenschutz in Niedersachsen
Adresse:	Medizinische Hochschule Hannover<br>Carl-Neuberg-Strasse 1<br>30625 Hannover	Adresse:	Postfach 221,<br>30002 Hannover oder<br>Prinzensreaße 5<br>30159 Hannover
Telefon:	0511 532 2555	Telefon:	0511 120 41576
E-Mail	weber.synia@mh-hannover.de	E-Mail	poststelle@lfd.niedersachsen.de

Datenschutz: Kontaktdaten des Sponsors der klinischen Studie in Marburg

Datenschutzbeauftragte/r		**Datenschutz-Aufsichtsbehörde**	
Name:	Die Präsidentin - Behördlicher Datenschutzbeauftragter Philipps-Universität Marburg	Name:	Der Hessische Datenschutzbeauf-tragte
Adresse:	Biegenstraße 10<br>35037 Marburg	Ad-resse:	Gustav-Stresemann-Ring 1<br>65189 Wiesbaden
Telefon:	06421-28 20	Telefon:	0611-140 80
Fax:	06421-28-22 065	Fax:	0611-140 8611
E-Mail:	datenschutz@uni-marburg.de	E-Mail	poststelle@datenschutz.hessen.de
Inter-net:	https://www.uni-marburg.de	Inter-net:	https://datenschutz.hes-sen.de/service/beschwerde

**Für die Datenverarbeitung Verantwortliche/r**	
ggf. Name	Präsidentin der<br>Philipps Universität Marburg
Adresse:	Biegenstr. 10<br>35037 Marburg
Telefon:	06421- 28 20
Fax:	06421-28 22500
E-Mail	info@uni-marburg.de
Internet:	https://www.uni-marburg.de

**Ich erkläre mich bereit,
an der oben genannten klinischen Prüfung
freiwillig teilzunehmen.**

Ein Exemplar der Patienten-Information und -Einwilligung (incl. Information bezüglich der DS-GVO) sowie die Versicherungsbedingungen habe ich erhalten. Ein Exemplar verbleibt im Prüfzentrum.

....
Name des Patienten in Druckbuchstaben

....
Datum Unterschrift des **Patienten**

Ich habe das Aufklärungsgespräch geführt und die Einwilligung des Patienten eingeholt.

....
Name des Prüfarztes/der Prüfärztin in Druckbuchstaben

....
Datum Unterschrift des aufklärenden **Prüfarztes/der Prüfärztin**

ANHANG zur VIGILung Patienten-Information und -Einwilligung

Mögliche Belastungen und Risiken durch Tacrolimus:

Infektionen und parasitäre Erkrankungen:
Wie bei anderen hochwirksamen Immunsuppressiva ist bei Patienten, die mit Tacrolimus behandelt werden, die Anfälligkeit für Infektionen häufig erhöht. Bereits bestehende Infektionen können sich verschlechtern. Infektionen können sich lokal oder im/am gesamten Körper manifestieren.
Fälle von BK-Virus-assoziierter Nierenentzündung durch BK-Viren und eine entzündliche Erkrankung der weißen Substanz des Gehirns durch JC-Viren (sog. progressive multifokale Leukoencephalopathie) wurden berichtet.

Gutartige, bösartige und unspezifische Neubildungen (einschl. Zysten und Polypen):
Bei Patienten, die mit Immunsuppressiva behandelt werden, erhöht sich das Risiko einer Tumorentwicklung. Es wurde über gutartige oder bösartige Tumore einschließlich Ebstein-Barr-Virus-assoziierter lymphoproliferativer Erkrankungen (Vorstufen einer Blutkrebserkrankung) und Hauttumoren unter Behandlung mit Tacrolimus berichtet.

Erkrankungen des Blutes und des Lymphsystems:
Häufig: Verminderung der roten Blutkörperchen (Blutarmut), Verminderung der weißen Blutkörperchen, Verminderung der Blutplättchen, Anstieg der Anzahl weißer Blutkörperchen, Veränderungen der roten Blutkörperchen

Gelegentlich: Blutgerinnungsstörungen, Verminderung der Gesamtzahl aller Blutkörperchen (weiße Blutkörperchen, rote Blutkörperchen, und Blutplättchen), Verminderung der neutrophilen Leukozyten (Unterart der weißen Blutkörperchen)

Selten: thrombotische thrombozytopenische Purpura (Hauteinblutungen aufgrund einer verminderten Anzahl von Blutplättchen), Hypoprothrombinämie (Verminderung eines Gerinnungsfaktors im Blut), thrombotische Mikroangiopathie (Blutgerinnsel in kleinen und kleinsten Blutgefäßen)

Häufigkeit nicht bekannt: Pure Red Cell Aplasia (Vollständiges Fehlen der roten Blutkörperchen aufgrund fehlender Produktion im Knochenmark), Agranulozytose (Mangel/Fehlen von Granulozyten, einer Unterart weißer Blutkörperchen), hämolytische Anämie (Verminderung roter Blutkörperchen durch vermehrten Abbau)

Erkrankungen des Immunsystems:
Unter der Anwendung von Tacrolimus wurden allergische Reaktionen beobachtet

Drüsen-Erkrankungen:
Selten: Vermehrte Körperbehaarung bei Frauen

Stoffwechsel- und Ernährungsstörungen:
Sehr häufig: Überzuckerung, Diabetes mellitus, erhöhte Kaliumwerte im Blut

Häufig: Magnesiummangel, Phosphatmangel, Kaliummangel, Kalziummangel, Natriummangel, Flüssigkeitsüberbelastung, Anstieg der Harnsäurewerte im Blut, verminderter Appetit, Übersäuerung, erhöhte Blutfettwerte, erhöhte Cholesterinwerte, andere Elektrolytstörungen

Gelegentlich: Flüssigkeitsmangel, Eiweißmangel im Blut, erhöhte Phosphatwerte im Blut, erniedrigte Blutzuckerwerte

Psychiatrische Erkrankungen:
Sehr häufig: Schlaflosigkeit

Häufig: Angsterscheinungen, Verwirrtheit und Desorientiertheit, Depression, depressive Verstimmung, affektive Störungen und Störungen des Gemütszustandes, Alpträume, Halluzinationen, Geisteskrankheiten

Gelegentlich: psychotische Störung

Erkrankungen des Nervensystems:
Sehr häufig: Zittrigkeit, Kopfschmerzen

Häufig: Krampfanfälle, Bewusstseinsstörungen, Missempfindungen, periphere Neuropathien (Schmerzstörungen in Händen und Füßen), Schwindelgefühl, Schreibstörung

Gelegentlich: Koma, Blutungen im Zentralnervensystem und Schlaganfall, Lähmungszustände und Lähmungen, Enzephalopathie (Schädigung des Gehirns), Sprech-und Sprachstörungen, Gedächtnis-Störungen

Selten: erhöhte Muskelspannung

Sehr selten: Muskelschwäche

Augenerkrankungen:
Häufig: verschwommenes Sehen, Lichtempfindlichkeit

Gelegentlich: Grauer Star

Selten: Blindheit

Häufigkeit nicht bekannt: Erkrankungen des Sehnervs

Erkrankungen des Ohrs und des Gleichgewichtsorgans:
Häufig: Ohrgeräusche (Tinnitus)

Gelegentlich: Hörschwäche

Selten: neurosensorische Taubheit

Sehr selten: eingeschränktes Hörvermögen

Herzerkrankungen:
Häufig: Koronare Herzgefäßkrankheit, Herzrasen

Gelegentlich: Herzrhythmusstörungen und Herzstillstand, Herzversagen, Herzmuskelerkrankung, Verdickung der Muskulatur der Herzkammern, Herzstolpern

Selten: Perikarderguss (Ansammlung von Flüssigkeit im Herzbeutel)

Sehr selten: Torsades de Pointes (spezielle Form der Herzrhythmusstörung mit besonders schnellen Herzschlägen)

Gefäßerkrankungen:
Sehr häufig: Bluthochdruck

Häufig: Blutungen, Blutgerinnsel-Bildungen und Embolien, sowie Durchblutungsstörungen, periphere Gefäßerkrankungen, Gefäßerkrankungen durch verminderten Blutdruck

Gelegentlich: Infarkt, tiefe Venenthrombose (Blutgerinnselbildung im Bereich der tiefen Venen), Kreislaufschock

Erkrankungen der Atemwege, des Brustraums und des Mediastinums:
Häufig: Atemnot, Erkrankungen des Lungengewebes, Pleuraerguss (Ansammlung von Flüssigkeit zwischen Rippenfell und Lunge), Rachenentzündung, Husten, Anschwellen und Entzündung der Nasenschleimhaut

Gelegentlich: Atemversagen, Asthma

Selten: akutes Atemnotsyndrom

Erkrankungen des Magen-Darm-trakts:
Sehr häufig: Durchfall, Übelkeit

Häufig: gastrointestinale Entzündung, Magen-Darm-Geschwür und Magen-Darm-Durchbruch, Blutungen aus dem Magen-Darm-Trakt, Mundschleimhaut-Entzündung und -Geschwür, Aszites (Wasseransammlung im Bauchfell), Erbrechen, Schmerzen im Magen-Darm-Bereich, Zeichen und Symptome einer gestörten Verdauung, Verstopfung, Blähungen und Aufgeblähtheit, lockerer Stuhl

Gelegentlich: Darmverschluss, akute und chronische Bauchspeicheldrüsenentzündung, gastroösophagealer Reflux (Rückfluss von Mageninhalt in die Speiseröhre), beeinträchtigte Magenentleerung

Selten: Unvollständiger Darmverschluss, Zystenbildung im Bereich der Bauchspeicheldrüse

Leber- und Gallenerkrankungen:
Häufig: Gallestau und Gelbsucht, Leberzellschaden und Leberentzündung, Gallengangs-Entzündung

Selten: Blutgerinnselbildung in der Leberarterie, mit Venenverschluss einhergehende Lebererkrankung

Sehr selten: Leberversagen, Gallengangs-Verschluss/Verengung

Erkrankungen der Haut und des Unterhautzellgewebes:
Häufig: Juckreiz, Ausschlag, Haarausfall, Akne, verstärktes Schwitzen

Gelegentlich: Hautentzündung, Lichtempfindlichkeit

Selten: Epidermolysis acuta toxica (Lyell-Syndrom) (Hautausschlag mit zum Teil großflächigen Ablösungen der oberen Hautschichten)

Sehr selten: Stevens-Johnson-Syndrom (Schwere Form eines Arzneimittel-bedingten Hautausschlages)

Skelettmuskulatur-, Bindegewebs- und Knochenerkrankungen:
Häufig: Gelenkschmerzen, Muskelkrämpfe, Schmerz in den Extremitäten, Rückenschmerzen

Gelegentlich: Gelenkerkrankungen

Selten: beeinträchtigte Beweglichkeit

Erkrankungen der Nieren und Harnwege:
Sehr häufig: Nierenfunktionsstörung

Häufig: Nierenversagen, akutes Nierenversagen, Oligurie, Tubulusnekrose (Gewebsuntergang in Teilbereichen der Niere), toxische Nephropathie (direkte Nierenzellschädigung durch das Medikament), Veränderungen des Harns, Symptome von Harnblase und Harnröhre

Gelegentlich: Verminderte oder fehlende Harnausscheidung, hämolytisch-urämisches Syndrom (spezielle Form des akuten Nierenversagens)

Sehr selten: Sonstige Nierenerkrankungen, blutige Blasenentzündung

Erkrankungen der Geschlechtsorgane und der Brustdrüse:
Gelegentlich: Unregelmäßige Periode und Gebärmutterblutungen

Allgemeine Erkrankungen und Beschwerden am Verabreichungsort:
Häufig: Kraft-/Energielosigkeit, fieberhafte Erkrankungen, Ödeme (Wassereinlagerungen), Schmerzen und Beschwerden, gestörtes Empfinden der Körpertemperatur

Gelegentlich: Organversagen mehrerer Organe, grippeartige Erkrankung, Temperatur-unverträglichkeit, Druckgefühl in der Brust, Zitterigkeit, Krankheitsgefühl

Selten: Durst, Sturz, Beklemmung in der Brust, Geschwüre

Sehr selten: Zunahme des Fettgewebes

Untersuchungen:
Häufig: Veränderungen der Leberwerte und Leberfunktion,

erhöhte Blutspiegel der alkalischen Phosphatase (bei Leber- und Knochenerkrankungen), Gewichtszunahme

Gelegentlich: erhöhte Amylasewerte (Hinweis auf mögliche Schädigung der Bauchspeicheldrüse), anormales EKG, anormale Herz- und Pulsfrequenz, Gewichtsverlust, erhöhte Laktatdehydrogenasekonzentration im Blut (allgemeiner Parameter bei Zellschädigung)

Sehr selten: anormales Echokardiogramm, QT-Verlängerung im Elektrokardiogramm (spezielle Form der EKG-Veränderung)

Verletzung, Vergiftung und durch Eingriffe bedingte Komplikationen:
Häufig: primäre Funktionsstörung des Transplantats

<u>Mögliche Belastungen und Risiken durch Mycophenolat-Mofetil</u>:

**Organsysteme**		**Nebenwirkungen**
Infektionen und parasitäre Erkrankungen	Sehr häufig	Blutvergiftung, Pilzinfektion des Darmes, Harnwegsinfektion, Herpes simplex Infektion, Gürtelrose
	Häufig	Lungenentzündung, Grippe, Infektion der Atemwege, Pilzinfektionen der Atemwege, Magen-Darm-Infektion, Pilzinfektionen, Infektion, Bronchitis, Rachenentzündung, Nasen-Nebenhöhlenentzündung, Pilzinfektion der Haut, vaginale Pilzinfektion, Schnupfen
Gutartige, bösartige und unspezifische Neubildungen (einschl. Zysten und Polypen)	Sehr häufig	–
	Häufig	Hautkrebs, gutartige Hauttumore
Erkrankungen des Blutes und des Lymphsystems	Sehr häufig	Verminderte Anzahl weißer Blutkörperchen, Verminderte Anzahl der Blutplättchen, Verminderte Anzahl der roten Blutkörperchen
	Häufig	Verminderte Anzahl aller Blutzellen, Anstieg der Anzahl der weißen Blutkörperchen
Stoffwechsel- und Ernährungsstörungen	Sehr häufig	–
	Häufig	Azidose, Erhöhte Kaliumwerte, Verminderte Kaliumwerte, Erhöhte Blutzuckerwerte, Verminderte Magnesiumwerte, Verminderte Kalziumwerte, Erhöhte Cholesterinwerte, Erhöhte Fettwerte, Verminderte Phosphatwerte, Erhöhte Harnsäurewerte, Gicht, Gewichtsverlust
Psychiatrische Erkrankungen	Sehr häufig	–
	Häufig	Erregungszustände, Verwirrung, Depression, Angstzustände, abnormes Denken, Schlaflosigkeit
Erkrankungen des Nerven-systems	Sehr häufig	–
	Häufig	Krampfanfälle, Erhöhter Muskeltonus, Zittrigkeit, ausgeprägte Müdigkeit, Muskelschwäche, Benommenheit, Kopfschmerzen, Empfindungsstörungen, Geschmacksstörung
Herzerkrankungen	Sehr häufig	–
	Häufig	Herzrasen
Gefäßerkrankungen	Sehr häufig	–
	Häufig	Erniedrigter Blutdruck, Bluthochdruck, Gefäßerweiterung
Erkrankungen der Atemwege, des Brustraums und Mediastinums	Sehr häufig	–
	Häufig	Wasseransammlung im Rippenfell, Kurzatmigkeit, Husten

**Systemorganklasse**		**Nebenwirkungen**
Erkrankungen des Gastro-intestinaltrakts	Sehr häufig	Erbrechen, Bauchschmerzen, Durchfall, Übelkeit
	Häufig	Magen-Darm-Blutungen, Bauchfellentzündung, Darmverschluss, Darmentzündung, Magengeschwür, Zwölffingerdarmgeschwür, Magenschleimhautentzündung, Entzündung der Speiseröhre, Entzündung der Mundschleimhaut,

		Verstopfung, Verdauungsstörung, Blähungen, Aufstoßen
Leber- und Gallenerkrankungen	Sehr häufig	–
	Häufig	Leberentzündung, Gelbsucht, Anstieg des Gelbfarbstoffes (Bilirubin)
Erkrankungen der Haut und des Unterhautzellgewebes	Sehr häufig	–
	Häufig	Verdickung der Haut, Hautausschlag, Akne, Haarausfall
Skelettmuskulatur-, Bindegewebs- und Knochenerkrankungen	Sehr häufig	–
	Häufig	Gelenkschmerzen
Erkrankungen der Nieren und Harnwege	Sehr häufig	–
	Häufig	Nierenversagen
Allgemeine Erkrankungen und Beschwerden am Verabreichungsort	Sehr häufig	–
	Häufig	Wassereinlagerungen, Fieber, Schüttelfrost, Schmerzen, Unwohlsein, Kraftlosigkeit
Untersuchungen	Sehr häufig	–
	Häufig	Erhöhte Leberwerte, erhöhte Nierenfunktionswerte im Blut, erhöhte Laktat-Dehydrogenase-Werte im Blut (Hinweis auf Zelluntergang), erhöhte Blut-Harnstoffwerte, erhöhte alkalische Phosphatase im Blut (Hinweis auf Leber- oder Knochenerkrankung), Gewichtsverlust

<u>Mögliche Belastungen und Risiken durch Mycophenolat-Natrium</u>:

Infektionen und parasitäre Erkrankungen	Sehr häufig	Virus-, bakterielle und Pilz-Infektionen
	Häufig	Infektionen der oberen Atemwege, Lungenentzündung
	Gelegentlich	Wundinfektionen, Blutvergiftung*, Knochenentzündung*
Gutartige, bösartige und unspezifische Neubildungen (einschl. Zysten und Polypen)	Gelegentlich	Papillom der Haut*, Basalzellkarzinom*, Kaposi-Sarkom*, lymphoproliferative Erkrankungen, Plattenepithelkarzinom*
Erkrankungen des Blutes und des Lymphsystems	Sehr häufig	Verminderung der weißen Blutkörperchen
	Häufig	Verminderung der roten Blutkörperchen, Verminderung der Blutplättchen
	Gelegentlich	Verminderung der Lymphozyten (Unterart der weißen Blutkörperchen)*, Verminderung der Neutrophilen (Unterart der weißen Blutkörperchen)*, Lymphknotenschwellung*
Stoffwechsel- und Ernährungsstörungen	Sehr häufig	Kalziummangel im Blut, Kaliummangel im Blut, Harnsäureanstieg im Blut
	Häufig	Kaliumanstieg im Blut, Magnesiummangel im Blut
	Gelegentlich	Gewichtsverlust, Anstieg der Blutfette, Diabetes mellitus*, Anstieg der Cholesterinwerte*, Phosphatmangel im Blut
Psychiatrische Erkrankungen	Sehr häufig	Angst
	Gelegentlich	Ungewöhnliche Träume*, Wahnvorstellungen*, Schlaflosigkeit*
Erkrankungen des Nervensystems	Sehr häufig	Schwindel, Kopfschmerzen
	Gelegentlich	Muskelzittern

Augenerkrankungen	Gelegentlich	Bindehautentzündung*, verschwommenes Sehen*
Herzerkrankungen	Gelegentlich	Herzrasen, Herzrhythmusstörung
Gefäßerkrankungen	Sehr häufig	Bluthochdruck
	Häufig	Verminderter Blutdruck
	Gelegentlich	Lymphozele (Lymphgefäßerkrankung)*
Erkrankungen der Atemwege, des Brustraums und Mediastinums	Häufig	Husten, Atemnot
	Gelegentlich	Erkrankung des Lungen-Gerüstes (Bindegewebe der Lunge), Lungenstauung*, Atemnot*, Wasseransammlung in der Lunge*
Erkrankungen des Gastrointestinaltraktes	Sehr häufig	Durchfall
	Häufig	Bauchblähung, Bauchschmerzen, Verstopfung, Verdauungsstörungen, Blähungen, Magenschleimhautentzündung, Übelkeit, Erbrechen
	Gelegentlich	Schmerzhafte Bauchdeckenspannung, Magen-Darmtrakt-Blutung, Aufstoßen, Mundgeruch*, Darmverschluss*, Lippengeschwüre*, Speiseröhrenentzündung*, Unvollständiger Darmverschluss*, Zungenverfärbung*, trockener Mund*, gastroösophageale Refluxkrankheit (Rückfluss von Mageninhalt in die Speiseröhre)*, Übermäßiges Zahnfleischwachstum*, Bauchspeicheldrüsenentzündung, Verstopfung des Ohr-Speicheldrüsen-ausfuhrgangs*, Magengeschwür*, Bauchfellentzündung*
Leber- und Gallenerkrankungen	Häufig	Anormale Leberfunktionstests
Erkrankungen der Haut und des Unterhautgewebes	Häufig	Akne, Juckreiz
	Gelegentlich	Haarausfall
Skelett-, Bindegewebs- und Knochenerkrankungen	Sehr häufig	Gelenkschmerzen
	Häufig	Muskelschmerzen
	Gelegentlich	Gelenkentzündung*, Rückenschmerzen*, Muskelkrämpfe
Erkrankungen der Nieren und Harnwege	Häufig	Anstieg des Blut-Kreatinins (Nierenfunktionswert)
	Gelegentlich	Blut im Urin*, renale tubuläre Nekrose (Nierengewebsschaden)*, Harnröhrenverengung
Erkrankungen der Geschlechtsorgane und der Brustdruse	Gelegentlich	Impotenz
Allgemeine Erkrankungen und Beschwerden am Verabreichungsort	Häufig	Kraftlosigkeit, Müdigkeit, Wasseransammlung in den Füßen/Beinen, Fieber
	Gelegentlich	Grippe-artige Erkrankungen, Wasseransammlung in den Füßen/Beinen*, Schmerzen, Muskelsteifheit*, Durst*, Schwächegefühl*
Verletzung, Vergiftung und durch Eingriffe bedingte Komplikationen	Gelegentlich	Prellungen*

*Der Fall trat nur bei einem einzigen (von 372 Patienten) auf.

Mögliche Belastungen und Risiken durch Azathioprin:

**Organsystem**		**Nebenwirkungen**
Infektionen und parasitäre Erkrankungen	Sehr häufig	Virale, Pilz-bedingte und bakterielle Infektionen bei Transplantatempfängern, die Azathioprin in Kombination mit anderen Immunsuppressiva erhalten
	Sehr selten	Nach Anwendung von Azathioprin zusammen mit anderen Immunsuppressiva wurde über Fälle von Progressiver multifokaler Leukenzephalopathie - PML (entzündliche Hirnerkrankung mit Beeinträchtigung der Sprache, des Fühlens und der Bewegung) berichtet
Gutartige, bösartige und unspezifische Neu-bildungen (einschließlich Zysten und Polypen)	Selten	Krebserkrankungen/Tumore, sog. lymphoproliferative Erkrankungen (Vorstufe von Blutkrebs), Hautkrebserkrankungen (schwarzer Hautkrebs und andere), Tumore des Bindegewebes (Kaposi-Sarkome und andere) und In-situ-Karzinom des Gebärmutterhalses, akute myeloische Leu- kämien und Myelodysplasien (Schädigungen des Knochenmarks)
Erkrankungen des Blutes und des Lymphsystems	Sehr häufig	Verminderung der Anzahl weißer Blutkörperchen
	Häufig	Verminderung der Anzahl der Blutplättchen
	Gelegentlich	Blutarmut
	Selten	Mangel/Fehlen von Granulozyten (weiße Blutkörperchen zur Infektabwehr), Verminderung aller Blutzellen, aplastische Anämie (Blutarmut durch Schädigung des Knochenmarks), megaloblastische Anämie (Blutarmut mit vermehrtem Auftreten von unreifen Knochenmarkszellen) und Versagen der Knochenmarksfunktion
	Sehr selten	Hämolytische Blutarmut durch Zerstörung der roten Blutkörperchen
Erkrankungen des Immunsystems	Gelegentlich	Überempfindlichkeitsreaktionen
	Sehr selten	Stevens-Johnson-Syndrom (schwere Form eines Arzneimittel-bedingten Hautausschlages), toxisch epidermale Nekrolyse (Ablösung der oberflächlichen Hautschichten)
Erkrankungen der Atemwege, des Brustraums und Mediastinums	Sehr selten	Reversible Entzündung des Lungengerüst-Gewebes
Erkrankungen des Gastrointestinaltrakts	Häufig	Übelkeit, bisweilen mit Erbrechen
	Gelegentlich	Bauchspeicheldrüsenentzündung
	Sehr selten	Dickdarmentzündung, Divertikulitis (Spezielle Form der Dickdarm-Entzündung) und Darm-Durchbruch
Leber- und Gallenerkrankungen	Gelegentlich	Gallestau
	Selten	Leberschäden
Erkrankungen der Haut und des Unterhautzellgewebes	Selten	Haarausfall
	nicht bekannt	Sweet-Syndrom (Fieber mit roten Knötchen und Ablagerungen im Gesicht und den Extremitäten, Sonnenlicht-Überempfindlichkeitsreaktion
Untersuchungen	Gelegentlich	Erhöhte Leberwerte.

Mögliche Belastungen und Risiken durch Prednisolon:

Infektionen und parasitäre Erkrankungen
Maskierung von Infektionen, Begünstigung von Virusinfektionen, Pilzinfektionen, von bakteriellen, parasitären sowie opportunistischen Infektionen,
Aktivierung einer Strongyloidiasis (Art einer Wurminfektion)

Erkrankungen des Blutes und des Lymphsystems
Mäßige Erhöhung der Anzahl weißer Blutkörperchen, Verminderte Anzahl bestimmter Arten weißer Blutkörperchen (sog. Lymphozyten, Eosinophile Granulozyten), erhöhte Anzahl aller Blutkörperchen

Erkrankungen des Immunsystems
Allergische Reaktionen (z. B. Hautausschlag), schwere allergische Reaktionen (mit beispielsweise Herzrhythmusstörungen, Verengung der Bronchien,
Blutdruckabfall- oder Bluthochdruck, Kreislaufkollaps, Herzstillstand,
Schwächung der Immunabwehr

Drüsen- Erkrankungen
Unterdrückung der Nebennierenfunktion und Induktion eines Cushing Syndroms (typische Symptome: Vollmondgesicht, Stammfettsucht und Wassereinlagerungen)

Stoffwechsel- und Ernährungsstörungen
Wassereinlagerungen durch verminderte Natriumausscheidung, vermehrte Kaliumausscheidung (Gefahr von Herzrhythmusstörungen),
Gewichtszunahme, diabetische Stoffwechsellage, Diabetes mellitus, erhöhte Cholesterinwerte und erhöhte Fettwerte,
Appetitsteigerung

Psychiatrische Erkrankungen
Depressionen, Gereiztheit, Euphorie, Antriebssteigerung,
Psychosen, Manie, Halluzinationen, Affektstörungen, Angstgefühle, Schlafstörungen,
Selbstmordgedanken

Erkrankungen des Nervensystems
Pseudotumor cerebri (Symptome eines Hirntumors, ohne dass ein Tumor vorliegt – z.B. Kopfschmerzen), Manifestation einer Epilepsie und Erhöhung der Anfallsbereitschaft bei manifester Epilepsie

Augenerkrankungen
Grauer Star, Grüner Star (Erhöhung des Augeninnendruckes), Verschlechterung der Symptome bei Hornhautgeschwür, Begünstigung viraler, Pilz-bedingter und bakterieller Entzündungen am Auge, verschwommenes Sehen

Gefäßerkrankungen
Bluthochruck, Erhöhung des Arteriosklerose- und Thromboserisikos, Gefäßentzündungen (auch als Entzugssyndrom nach Langzeittherapie), erhöhte Brüchigkeit kleinster Gefäße

Erkrankungen des Gastrointestinaltraktes
Magen-Darm-Geschwüre, Magen-Darm-Trakt- Blutungen,
Bauchspeicheldrüsen-Entzündung

Erkrankungen der Haut und des Unterhautzellgewebes
Schwangerschafts-Streifen, Verdünnung der Haut, Sichtbare Erweiterung der Kapillargefäße, stichartige Einblutungen, flächige Hautblutungen, Vermehrung der Körperbehaarung,
Akne, rosazea-artige Hautentzündung (ähnlich einer Akne, hauptsächlich im Nasen-Mund-Bereich), Änderungen der Hautpigmentierung

Skelettmuskulatur-, Bindegewebs- und Knochenerkrankungen
Muskelschwund und -schwäche, Muskelschmerzen,
Osteoporose (dosisabhängig, auch bei nur kurzzeitiger Anwendung möglich), nicht-entzündlicher Gewebe-Untergang im Bereich des Knochens, Sehnenbeschwerden, Sehnenentzündung, Sehnenrupturen und Fettansammlung im Rückenmarkskanal, Wachstumshemmung bei Kindern
