# Supplementary material for: Viral load-guided immunosuppression after lung transplantation (VIGILung)—study protocol for a randomized controlled trial
Source: Trials. 2021 Jan 11;22:48. doi: 10.1186/s13063-020-04985-w (PMC7798016; doi:10.1186/s13063-020-04985-w)
Supplement: Supplementary file 2 — Additional file 2. Translation of the funding information [file 13063_2020_4985_MOESM2_ESM.pdf]

Deutsche
Forschungsgemeinschaft

Lebenswissenschaften 3:
Medizin

Kennedyallee 40
53175 Bonn

Dr. Katja Großmann

Telefon: +49 228 885-2565
Telefax: +49 228 885-2777
katja.grossmann@dfg.de

Fragen beantwortet:
Christiane Krämer

Telefon: +49 228 885-2564
Telefax: +49 228 885-2777
christiane.kraemer@dfg.de
www.dfg.de

Deutsche Forschungsgemeinschaft · 53170 Bonn

Herrn
Professor Dr. Jens Gottlieb
Medizinische Hochschule Hannover
Zentrum Innere Medizin
Klinik für Pneumologie
Carl-Neuberg-Straße 1
30625 Hannover

GZ: GO 2800/1-1 AOBJ: 650839 17.07.2018 Rul

Sehr geehrter Herr Professor Gottlieb,

die Deutsche Forschungsgemeinschaft bewilligt Ihnen und Ihrer Hochschule entsprechend Ihrem Antrag mit dem Titel "Viral load guided Immunosuppression after Lung Transplantation (VIGILung)", den Sie im DFG-Programm "Klinische Studien" gestellt haben, Mittel bis zur Höhe von 825.201 Euro zuzüglich 181.500 Euro Programmpauschale für 36 Monate.

Die Mittel werden als Drittmittelbewilligung zur Verfügung gestellt. Die Abrechnung erfolgt im Drittmittelverfahren. Es handelt sich um eine flexibilisierte Förderung im Sinne der Ziffer 6 der Verwendungsrichtlinien.

Im Einzelnen werden Ihnen für die Module - Basismodul - die folgenden Mittel bewilligt:

	Anz.	Vol.	Dauer	Euro
**GO 2800/1-1**				
**Professor Dr. Jens Gottlieb**			**36 Mon.**	
durch DFG finanziert				**1.006.701**
**Personalmittel**				**269.700**
Ärztliche wiss. Mitarbeiterin/Ärztlicher wiss. Mitarbeiter	1	100%	12,7 Mon.	98.400
Postdoktorandin/Postdoktorand und Vergleichbare	1	100%	8,3 Mon.	48.500
Postdoktorandin/Postdoktorand und Vergleichbare	1	100%	1 Mon.	5.800

DFG

Postdoktorandin/Postdoktorand und Vergleichbare	1	100%	4,5 Mon.	26.200
Sonstige wiss. Mitarbeiterin/Sonstiger wiss. Mitarbeiter	1	100%	16,5 Mon.	75.900
Sonstige wiss. Mitarbeiterin/Sonstiger wiss. Mitarbeiter	1	100%	3,3 Mon.	14.900
**Sachmittel**				**555.501**
**Investitionsmittel**				-
**Programmpauschale**				**181.500**

Dem darüber hinausgehenden Antrag konnte leider nicht entsprochen werden.

Mittel zur Vorbereitung der Studiendurchführung (Erstellung des Studienprotokolls, Abschluss einer Patientenversicherung und Wegeversicherung, Unterlagen für die Zulassungsbehörden, vorbereitende Treffen) können bis zu einer Höhe von 100.000 Euro sofort abgerufen werden.

Weitere Mittel können erst in Anspruch genommen werden, wenn folgende Nachweise erbracht worden sind:

- Zustimmendes Votum der zuständigen Ethikkommission unter Beteiligung der zuständigen Ethikkommissionen aller rekrutierenden Zentren
- Vorlage der von der Ethikkommission gebilligten Version des Studienprotokolls
- Vom Vertreter der medizinischen Einrichtung unterzeichnete Erklärung zur Übernahme der Verpflichtung zur Einhaltung der Guten Klinischen Praxis
- Registrierung der Studie in einem öffentlichen Register (z. B. http://www.germanctr.de, www.controlled-trials.com, www.clinicaltrials.gov)
- Hinterlegung des Studienprotokolls in einem öffentlich zugänglichen Register oder Veröffentlichung in einem peer-reviewed Journal
- Einrichtung eines unabhängigen Datenüberwachungsgremiums.

Sind die oben genannten Nachweise erfolgt, können die bewilligten Mittel in den folgenden Tranchen abgerufen werden:

- Bis zu 10 % der insgesamt bewilligten Mittel sofort
- Bei Nachweis des Einschlusses des 1. Studienpatienten (ca. 6 Monate nach Bewilligung) können maximal weitere 25 % der insgesamt bewilligten Mittel abgerufen werden
- Bei Nachweis des Einschlusses der ersten 25 % der Studienpatienten (ca. 18 Monate nach Bewilligung) können maximal weitere 20 % der insgesamt bewilligten Mittel abgerufen werden
- Bei Nachweis des Einschlusses der Hälfte der Studienpatienten (ca. 26 Monate nach Bewilligung) können maximal weitere 20 % der insgesamt bewilligten Mittel abgerufen werden
- Bei Nachweis des Einschlusses von 75 % der Studienpatienten (ca. 35 Monate nach Bewilligung) können die restlichen Mittel abgerufen werden.

Das Erreichen der oben genannten Meilensteine ist zu den gegebenen Zeitpunkten schriftlich zu bestätigen..

Zwölf Monate nach Erhalt des Bewilligungsschreibens ist unaufgefordert ein Zwischenbericht vorzulegen. Anschließend sind immer halbjährlich weitere Zwischenberichte einzureichen. Bitte richten Sie sich bei der Erstellung der Zwischenberichte nach unseren Vorgaben auf der DFG Homepage (siehe Programm Klinische Studien). Die Zwischenberichte dienen der Verlaufskontrolle der bewilligten Studie und können bei Nicht-Erreichen der Meilensteine der Begutachtungsgruppe zur erneuten Diskussion vorgelegt werden.

Die Mittel für *case payments* (Patientenfallgelder) sind fallbezogen an die beteiligten Studienzentren weiterzugeben. Höhe, Anzahl und Weitergabe der *case payments* sind zu dokumentieren.

Werden Patienten im Ausland rekrutiert, so können bis zu 20% der *case payments* ohne Rücksprache mit der Deutschen Forschungsgemeinschaft an ausländische Studienzentren weitergeleitet werden.
Die darüberhinausgehenden Mittel für die Rekrutierung im Ausland wurden im Rahmen der Begutachtung von der Begutachtungsgruppe Klinische Studien geprüft und für begründet erachtet.

Die DFG behält sich die Möglichkeit zur Durchführung eines Audits der Studie vor.

Kosten für Probandenversicherungen werden nur dann von der DFG übernommen, wenn die betreffende Forschungseinrichtung eine Erklärung abgibt, wonach sie entweder

- zum Abschluss dieser Versicherungen berechtigt ist, oder aber
- um eine entsprechende Ausnahmegenehmigung des zuständigen Finanzministeriums nachsuchen wird. Die Ausnahmegenehmigung muss bis zur Schlussabrechnung des geförderten Projekts vorliegen. Die DFG ist über die Beantragung einer solchen Ausnahmegenehmigung zu unterrichten.

Sofern im Rahmen des Forschungsvorhabens Auslandsreisen durchgeführt werden, so sind die Sicherheitshinweise und Reisewarnungen des Auswärtigen Amtes zu berücksichtigen. Für Risiken, die sich aus einem Auslandsaufenthalt ergeben, kann die DFG keine Verantwortung übernehmen.

Hinsichtlich der Programmpauschale beachten Sie bitte Ziffer 3.6 der Verwendungsrichtlinien (DFG-Vordruck 2.00 – 03/17).

Die DFG geht davon aus, dass bei der Planung und Durchführung von Forschung an Menschen, an identifizierbarem menschlichen Material und an identifizierbaren Daten die vom Weltärztebund (WMA - World Medical Associ-

ation) im Juni 1964 verabschiedete Deklaration von Helsinki (Originaltitel: DECLARATION OF HELSINKI -Ethical Principles for Medical Research Involving Human Subjects) in der jeweils gültigen Fassung beachtet wird.

Die Stellungnahmen zu Ihrem Antrag liegen in anonymisierter Form bei.

Bei Fragen zur finanziellen Abwicklung der bewilligten Mittel wenden Sie sich bitte unter Angabe des Geschäftszeichens GO 2800/1-1 und des dazugehörigen Abrechnungsobjektes 650839 an den Bereich Finanzielle Umsetzung von Förderentscheidungen, E-Mail FIN2@dfg.de.

Die beigefügten Verwendungsrichtlinien (DFG-Vordruck 2.00 – 03/17) sind Bestandteil dieser Bewilligung.

Ihre Hochschule wird mit einem Schreiben gleichen Datums zum obigen Geschäftszeichen ebenfalls über den Umfang der Bewilligung informiert.

Sie werden gebeten, die Vertrauensdozentin Ihrer Hochschule für Angelegenheiten der Deutschen Forschungsgemeinschaft Frau Professorin Dr. Christine Falk, Carl-Neuberg-Straße 1, 30625 Hannover, von dieser Bewilligung zu unterrichten.

Mit Annahme dieser Bewilligung verpflichten Sie sich, gleich nach Abschluss Ihres Projekts über die Ergebnisse zu berichten (siehe "Leitfaden für Abschlussberichte" in den beigefügten Verwendungsrichtlinien), wir haben dafür als Termin vorläufig den 01.07.2021 notiert.
Wenn Sie jedoch einen Fortsetzungsantrag zu diesem Projekt stellen, so berichten Sie bitte nur darin unter „Eigene Vorarbeiten" über Ihre bisherige Arbeit.
Abschlussberichte werden von der DFG nur anerkannt, wenn die Studienergebnisse innerhalb von zwei Jahren nach Projektende veröffentlicht wurden.

Projektergebnisse, die aus mit DFG-Mitteln finanzierten Projekten resultieren, müssen in geeigneter Art und Weise der Allgemeinheit zugänglich gemacht werden. Die Veröffentlichungen müssen einen Hinweis auf die DFG-Förderung enthalten. Hierbei sind ausschließlich die in Ziffer 12.1 der Verwendungsrichtlinien genannten Schreibweisen zu verwenden.

Ihre Projektnummer lautet 329015138.

Die zur Bearbeitung Ihres Antrags erforderlichen Daten wurden von der DFG elektronisch gespeichert und verarbeitet. Zu der hier bewilligten Fördermaßnahme werden Adress- und Kommunikationsdaten zur Person (Name, Institution und Ort, Telefon, Fax, E-Mail, www-Homepage) sowie inhaltserschließende Angaben (z. B. Thema, Zusammenfassung, Schlagwörter, fachliche Zu-

ordnung, DFG-Verfahren, Förderzeitraum, Auslandsbezug) in der Projektdatenbank GEPRIS (vgl.: http://www.dfg.de/gepris/) veröffentlicht. Wenn Daten anders als in der Ihrem Antrag entnommenen Form angegeben werden sollen oder keine elektronische Publikation erfolgen soll, teilen Sie uns dies bitte innerhalb einer Frist von vier Wochen schriftlich mit.

Die Deutsche Forschungsgemeinschaft wünscht Ihnen für Ihre Arbeit guten Erfolg.

Mit freundlichen Grüßen

Katja G.

Dr. Katja Großmann

Stellungnahmen zum Antrag

Projekt: "Viral load guided Immunosuppression after Lung Transplantation (VIGILung)" (GO 2800/1-1)

Antragsteller: Herr Professor Dr. Jens Gottlieb, Hannover

Erstes klinisches Gutachten:
“This full proposal on immunosuppression individualisation in lung transplantation based on TTV load postulates that dose adjustments of immunosuppressants according to viral load may optimize the efficacy and safety balance in lung transplantation. This project suggests to use TTV load as a kind of surrogate pharmacodynamic parameter reflecting the burden of immunosuppression. As it is very difficult to assess or measure the impact of immunosuppressive drugs' morbidity, tacrolimus nephrotoxicity has been chosen to quantify, by means of GFR, one of the benefits of immunosuppression reduction. According to the working hypothesis, such a single parameter should translate the benefits of drug therapy individualization with an adequate ratio between organ rejection/dysfunction and comorbidities. One of the concerns on this approach is that in the context of multitarget therapy, immunosuppression adjustments will include not only tacrolimus dose variations but also MMF. In this regard, it might be very important to prespecify a homogeneous induction therapy, trying to avoid discretionality, especially taking into account that depletive therapies directly impact on viral replication and load. The project represents a nice opportunity to evaluate anti-viral specific T cell responses, which in turn might correlate with specific viral loads.

The assumptions made on the impact of tacrolimus exposures on renal function entail some risk for the project. In this regard, consider assessing the Co tacrolimus AUCs overtime in the two groups for comparisons, which in turn might correlate with efficacy/safety parameters on top of the impact on GFR. Consider if such approach may help to reduce the risk of the project.

This project is very pertinent taking into account the paucity of validated parameters to evaluate drug-dosing refinements in organ transplantation after 3 decades of the introduction of CNIs. In lung transplantation, the suggested approach might contribute to the development of precision medicine in lung transplant recipients population, one with the highest risk in organ transplantation.

According to previous considerations, the funding priority of this project might be medium/high. Despite the risks described, if the project meets the end-points, it may change the paradigm on immunosuppression management in solid organ transplantation beyond the limitations of the traditional PK measurements. In an era of sophisticated biomarker development, such a relatively easy approach might disseminate in clinical practice.
According to the sample size estimated and the multicentre nature of the project the asked financial support seems reasonable, and the applicants may consider reallocating some funding for immune mechanistic studies.”

Zweites klinisches Gutachten:

„Starting hypotheses

General evidence in the proposal

The underlying hypothesis of the trial is that Torque-Teno-Virus (TTV) viral load can be used to guide conventional, standard immunosuppression, following lung transplantation. The hypothesis that will be formally tested is that this guided immunosuppression might reduce known side effects of immunosuppression, particularly renal toxicity. Since this is a proof of principle study, there is no direct evidence so far to support these hypotheses. However, there are well published retrospective data, mainly of the Vienna group participating in this trial, that strongly suggest that TTV viral load can be used to guide immunosuppression. The retrospective studies were performed in renal as well as lung transplant recipients.

Overall, the existing evidence convincingly supports the trial rationale.

Effect size of the experimental intervention

As primary end point the study will investigate the effect of the TTV guided immunosuppression on kidney function, as shown by changes in glomerular filtration rate (GFR). There is ample evidence that renal function deteriorates after lung transplantation due to the indispensable use of immunosuppressive drugs, especially Tacrolimus. The reduction in renal function under conventional immunosuppression is so strong that an effect in the investigative arm can be expected.

The trial has a significant clinical relevance for all transplant patients, not only lung allograft recipients

Diagnostic/prognostic trial

Assessment of the TTC viral load has been established in the Vienna group, which has a well published history of this test system. The other clinical assessments, including rejection diagnostics, are performed according to the standard of care in highly referenced transplant centers.

From the proposal it remains unclear were and at which interval the actual Tacrolimus drug levels are being determined, at a primary care center or the transplant centers? This seems important since the TTV viral load only determines the aspired target level for the following time period. Therefore the timely and accurate calibration of the respective drug level is critical. Surely the investigators will have considered this point, but it is not stated in the proposal.

Concept for clinical or epidemiological action/further steps in research

This is a pilot study. If the outcome shows that TTV viral load can be used to guide immunosuppression the results will have an impact on other organ transplant programs as the investigators point out. Even a negative outcome would be of importance.

Significance of the topic/ethical considerations

Novelty

So far only retrospective data exist, correlating TTV viral load with immunosuppression and rejection following solid organ transplantation. This will be the first prospective trial investigating the feasibility of TTV viral load guided immunosuppression, and one of the first prospective trials overall using immunological or viral surrogate markers to guide immunosuppression.

The question addressed in the trial is novel.

Potential impact of relieving the burden of disease and/or improving human health

A general shortcoming in transplantation medicine is the lack of prospective trials. Therefore, this proposal is an important initiative that is undoubtedly of major interest to the entire transplant

community. Due to the dramatic organ shortage, the still unsatisfactory long term graft survival, and the side effects of the live long immunosuppression the attempt of guiding and optimizing immunosuppression seems worthwhile.

Ethics

This trial uses standard, in label use immunosuppression. TTV viral load guided immunosuppression is expected to reduce overall Tacrolimus dosage and subsequently renal toxicity, which would be of major benefit for the allograft recipients. On the other hand, there is a potential risk of higher rejection rates in the test arm. However, the trial uses a 3-monthly thorough work up to detect early signs of rejection. Rejection rates are a secondary outcome and will be monitored by the DSMB.

Therefore, the trial is ethically acceptable.

Design aspects

The study uses a two-arm parallel assignment, a simple but robust design that seems adequate for such a pilot study. The inclusion/exclusion criteria are based on previous published data with in lung allograft recipients. However, the results can be exploited for other organ transplant programs. The outcome measure for the primary and secondary endpoints seems reasonable, as well as the statistical considerations.

Feasibility

This pilot study uses standard, in label use immunosuppression. The TTV test is established in the Vienna group and well referenced. All participating centers, especially Vienna and Hannover are internationally high ranking lung transplant programs. The quality and safety measures and follow up required in the study in the post-transplant phase is the expertise of the respective centers. Vienna and Hannover have stable, high numbers of lung transplants per year. The estimated participation, compliance and dropout rates seem conservatively calculated. All centers have a long experience with this special patient cohort, including study participation. Hence the recruitment rate seems feasible.

Qualifications of applicant(s)/trial management

Team of investigators

The PI and the participating centers have a long track record in this respective field, and are internationally recognized in the field of lung transplantation. The other key participants have an equally strong expertise in their respective field. The PI and the leading Hannover center have also proven that it can successfully conduct investigator initiated trials that were published in high ranking transplant journals.

Trial coordination and advisory bodies

The trial coordination is and data management is transferred to a Coordinating Centre for Clinical Trials (KKS Marburg). Here the proposed financial support for this institution seems very generously calculated, for this type of trial.

Due to a potentially increased rejection rate in the study arm, a data and safety monitoring board (DSBM) is certainly necessary required. This DSMB is adequately defined in the proposal.

Commercial exploitation

There is no economic benefit from the potential trial results to be expected for a company. The test for the TTV viral load assessment is published.

Funding recommendation/comments

I would recommend funding of the proposed trial with a high priority.

However, the cost calculations for the KKS Marburg seem rather high for this type of study, with redundancy in management positions.
Also it is unclear whether the follow up investigations will be covered by the German or Austrian health system or whether all investigations have to be covered by the case payment."

Biostatistisches Gutachten:
"Gegenstand des Antrags ist eine klinische Studie zur Steuerung der Immunsuppression bei Patienten nach Lungentransplantation. Während das bisherige Standardvorgehen eine feste Vorgabe des Dosis des immunsuppressiven Medikaments mit alleiniger Korrektur durch therapeutisches Drug-Monitoring ist, soll in dieser Studie eine Anpassung der Dosis anhand der ermittelten Torque-Teno-Viruslast vorgenommen werden, um so Unter- sowie Überdosierungen mit den jeweiligen schwerwiegenden Konsequenzen zu vermeiden. Erste vielversprechende Erfahrungen hierzu liegen vor. Im Rahmen dieser Studie soll die neue Methode aber erstmals in einer prospektiven, multizentrischen, randomisierten Studie mit der Standardtherapie verglichen werden. Unter den drei beteiligten Zentren befindet sich auch die Abteilung Thoraxchirurgie des Universitätsklinikums Wien, wo Grundlagen dieser neuen Anpassung entwickelt wurden.

Die Studie könnte einen großen Einfluss auf die zukünftige Behandlung von Patienten nach Lungentransplantation und auch nach anderen Organtransplantationen haben und ist sehr innovativ. Wie bereits bemerkt, sind die Antragsteller ausgewiesene Experten auf dieser Strecke.

Das Studiendesign ist einfach, aber der Fragestellung angemessen. Die Hypothesen sind klar formuliert. Die Ein- und Ausschlusskriterien sind gut begründet, ebenfalls der verwendete primäre Endpunkt. Die geplante permutierte Blockrandomisation mit zwei Strata ist beim gegebenen Stichprobenumfang angemessen.

Gegenüber der ersten Antragsstufe haben die Antragsteller die Begründung der Fallzahl verbessert. Insbesondere wurde das angenommene Effektmaß durch zwei neue Studien belegt. Die geplanten Fallzahlen scheinen für die drei Zentren auch realisierbar zu sein.

Die geplante Analysestrategie für den primären Endpunkt mittels U-Test ist grundsätzlich akzeptabel. Sie sollte aber durch eine Sensitivitätsanalyse mit geeigneter Einbeziehung der Stratifizierungsfaktoren als Kovariablen ergänzt werden, wie es schon für die sekundären Endpunkte beschrieben wurde. Die im Gutachten für die erste Antragsstufe angeforderte Erklärung zum Umgang mit fehlenden Werten blieb leider letztlich sehr allgemein mit einem Verweis auf ein Lehrbuch über den Umgang mit fehlenden Werten.

Diese letzte Kritik beeinflusst nicht die positive Gesamteinschätzung der Studie. Diese Frage der fehlenden Werte bzw. die drop-out-Problematik muss dann im Studienprotokoll adäquat und insbesondere konkret behandelt werden. Daher unterstütze ich die Förderung dieser Studie.

Die beantragten Mittel kann ich nur bedingt einschätzen. Das betrifft insbesondere die Frage, ob die für die Berechnung der Fallpauschale aufgeführten Posten tatsächlich alle als studienbedingt anzusehen sind. Die restlichen Mittel scheinen angemessen."

<u>Stellungnahme der Begutachtungsgruppe Klinische Studien:</u>

„Zu diesem Antrag auf Förderung einer klinischen Studie liegen drei Gutachten vor. Nach Meinung des ersten Gutachtenden ist die Studie hoch relevant und könnte zu einen Paradigmenwechsel im Immunosuppressions-Management führen. Die Vorschläge dieses Gutachtenden zur Endpunkterhebung sollen dem Antragsteller aus Sicht der Begutachtungsgruppe „Klinische Studien" als positiver Denkanstoß dienen. Der zweite Gutachtende sieht die Studie ebenfalls für neuwertig und klinisch hoch relevant ein. Das Studiendesign, die Vorarbeiten sowie die Expertise des Studienteams werden gelobt. Aus Sicht des biometrischen Gutachtenden ist die Planung und Auswertung der Studie überzeugend. Die angeführten Hinweise zu drop-out-Problematik sprechen aus Sicht des Gutachtenden nicht gegen eine Förderung.

Die Begutachtungsgruppe „Klinische Studien" schließt sich der positiven Einschätzung der Gutachtenden an. Es handelt sich um eine spannende Studienidee, die von einen ausgewiesenen Kliniker geleitet werden soll. Die eigebundenen Zentren in Deutschland und Österreich erscheinen für eine erfolgreiche Durchführung der Studien sehr gut geeignet. Die Finanzierung der Rekrutierungskosten in Österreich sollen von der Deutschen Forschungsgemeinschaft getragen werden. Da jedoch die Höhe der beantragten Personalmittel zum Teil nicht gerechtfertigt erscheint, schlägt die Begutachtungsgruppe eine Bewilligung unter Kürzung der Personalmittel in Höhe von rund 100.000 Euro vor. Demzufolge werden zwei Postdoktorandenstellen (100%, 9 Monate und 100% 8,3 Monate) abgelehnt."
