# Supplementary material for: Viral load-guided immunosuppression after lung transplantation (VIGILung)—study protocol for a randomized controlled trial
Source: Trials. 2021 Jan 11;22:48. doi: 10.1186/s13063-020-04985-w (PMC7798016; doi:10.1186/s13063-020-04985-w)
Supplement: Supplementary file 3 — Additional file 3. Translation of the Ethics committee’s vote of Hannover Medical School [file 13063_2020_4985_MOESM3_ESM.pdf]

MHH
Medizinische Hochschule Hannover

Ethikkommission
Vorsitzender:
Prof. Dr. Stefan Engeli

Sekretariat:
Marion Lange
Telefon: 0511 532-3443
Liane Höft
Telefon: 0511 532-9812
Fax: 0511 532-16 3443
ethikkommission@mh-hannover.de

Carl-Neuberg-Straße 1
30625 Hannover
Telefon: 0511 532-0
www.mh-hannover.de

MHH Ethikkommission OE 9515
30623 Hannover

KKS Marburg
Standort Marburg
Frau Dr. Karin Weide
Karl-von-Frisch-Str. 4
35043 Marburg

19.11.2019/MLa

Nr. 8579_AMG_mono_2019
EudraCT-Nr. 2019-001770-29
Viral load guided Immunosuppression after Lung Transplantation - An open-label, randomized, controlled, parallel-group, multicenter trial (VIGILung) (Prüfplan-Code: KKS-256)

Sehr geehrte Frau Dr. Weide,

die Ethik-Kommission hat über Ihren o. g. Antrag in der Sitzung am 14.08.2019 und nach Eingang der überarbeiteten Unterlagen über diese Studie abschließend beraten. Die Ethikkommission erteilt eine zustimmende Bewertung, da Versagensgründe nach dem Arzneimittelgesetz (AMG) nicht vorlagen. Die klinische Prüfung ist ärztlich vertretbar, ein Nutzen für die Heilkunde ableitbar.

Die Ethikkommission weist darauf hin, dass die ärztliche und juristische Verantwortung beim Prüfarzt verbleibt.

An der abschließenden Beratung und Beschlussfassung haben die unten aufgeführten Mitglieder der hiesigen Ethikkommission teilgenommen. Es wird bestätigt, dass Mitglieder der Ethikkommission, die an der o. g. Studie beteiligt sind, nicht an der Abstimmung teilgenommen haben.

Die Ethikkommission gibt folgende allgemeine Hinweise:

1. Auf die Einhaltung einschlägiger Gesetze und Rechtsvorschriften wird hingewiesen. Die nach Rechtslage notwendigen Unterrichtungen (u. a. Prüfplanänderungen, entsprechende Zwischenfallereignisse, neue Datenlage, Nachmeldung von Prüfzentren, Jahresbericht, Abschlussbericht) sind unverzüglich vorzulegen.
2. Die Ethikkommission bestätigt, dass sie auf Grundlage nationaler Gesetze, Vorschriften sowie der GCP/ICH-Richtlinie arbeitet.
3. Eine Kopie dieser Stellungnahme wird der zuständigen Behörde zugeleitet.
4. Gegen die vorliegende Stellungnahme kann innerhalb von einem Monat nach Bekanntmachung Widerspruch erhoben werden. Der Widerspruch ist schriftlich bei der Geschäftsstelle der federführenden Ethikkommission zusammen mit einer Begründung einzureichen.

Die Ethikkommission weist darauf hin, dass die ärztliche und juristische Verantwortung bei den jeweiligen Prüfärzten verbleibt.

Datenschutzrechtliche Aspekte von Forschungsvorhaben werden durch die Ethikkommission grundsätzlich nur kursorisch geprüft. Dieses Votum / diese Bewertung ersetzt mithin nicht die Konsultation des zuständigen Datenschutzbeauftragten.

Mit den besten Grüßen

Prof. Dr. Stefan Engeli
Vorsitzender

Nachrichtlich:
Ethikvotum@bfarm.de

Das Votum ist gültig für folgende Prüfstelle und die Prüfärzte:

- Prof. Dr. Jens Gottlieb, Dr. Mark Greer, Klinik für Pneumologie, Medizinische Hochschule Hannover

Vorgelegen zur Begutachtung haben:

	Inhalt	Datum/Version	Verzeichnisname (.pdf)
1.	Bestätigungsschreiben EudraCT-Nummer	15.04.2019	VIGILung_Application for EudraCT Number
2.	Begleitschreiben	25.07.2019	2019-07-25_VIGILung_Brief an fEK
3.	Prüfplan incl. Unterschriften	25.07.2019/V02F	01_VIGILung_Study-Protocol_V02F_2019-07-25<br>02_VIGILung_Study-Protocol_Unterschriften_V02F_2019-07-25
4.	Deutsche Synopse	24.07.2019/V01F	VIGILung_Study Synopsis_Deutsch_V01F_2019-07-24
5.	Antragsformulare (Modul 1, Modul 2)	18.06.2019/V02F	01_VIGILung_V02F_2019-001770-29 DE 20190618 CTA.xml
		18.06.2019/V02F	02_VIGILung_V02F_2019-001770-29 DE 20190618 CTA PDF Form_EK_mU
		25.07.2019	03_2019-07-25_VIGILung_Modul 2 - April2019
6.	Patienteninformation und Einwilligungserklärung	V01F/25.07.2019	VIGILung_ Pat Info+Einwilligung_V01F_2019-07-25
7.	Fachinformationen (SmPCs)	März 2018	Fachinformation-CellCept(Mycophenolat-Mofetil)Tabletten-März-2018

September-2017 Fachinformation-DecortinH(Prednisolon)-September-2017
August-2018 Fachinformation-Imurek(Azathioprin)-August-2018
August-2018 Fachinformation-Myfortic(Mycophenolate-Natrium)-August-2018
Februar-2019 Fachinformation-Prograf(Tacrolimus)Hartkapseln-Februar-2019

8. Qualifikationsunterlagen
Hannover (Zentrum 01)
01_1_Angaben Prüfer und FD_Prof. Gottlieb
01_1_CV_Prof. Gottlieb_2019-04-24 GCP-Zertifikate_Prof. Gottlieb_2008-2009
01_2-6_Prüfzentrum Hannover
01_7_Angaben Stellvertreter und FD_Dr. Greer
01_7_CV_Dr. Greer_2019-05-10 GCP-Zertifikate_Dr. Greer_2010-2019

9. Versicherungsunterlagen
01_Probandenversicherung_Bestätigung HDI_VB 11042019103
02_Probandenversicherung_VB_404-U.1991.16.pdf_AMG_JV
03_Wegeunfallversicherung_Versicherungsschein
04_Wegeunfallversicherung_Versicherungsbedingungen_Scan

10 Bewilligungsbescheid DFG 17.07.2018 2018-07-17_DFG Bewilligung

11. Sponsorerklärung 15.04.2019 01_9_GCP_Erklärungen des Sponsors

12. Sponsorvollmacht 31.01.2006 Sponsorvollmacht-KKS_2006-01-31

13. Checkliste AMG 25.07.2019 2019-07-25_VIGILung_ChecklisteAMG05102012

14. Liste der Unterlagen V01F/25.07.2019 VIGILung_Liste der Unterlagen V01F_2019-07-25

11.11.2019

	Inhalt	Datum/Version	Verzeichnisname
1.	Begleitschreiben	11.11.2019	01_2019-11-11_VIGILung_Antwort an fEK
2.	Prüfplan incl. Unterschriften	05.11.2019/V03F	02_VIGILung_Study-Protocol_V03F_2019-11-05<br>02_VIGILung_Study-Protocol_V03F_2019-11-05_mÄ<br>02_VIGILung_Study-Protocol_Unterschriften_V03F_2019-11-05
3.	Antragsformular (Modul 1)	11.11.2019/V03F	03_VIGILung_V03F_2019-001770-29 DE 20191111 CTA
		11.11.2019/V03F	03_VIGILung_V03F_2019-001770-29 DE 20191111 CTA PDF Form_EK_mU
4.	Patienteninformation und Einwilligungserklärung	06.11.2019/V02F	04_VIGILung_PatInfo+Einwilligung_V02F_2019-11-06<br>04_VIGILung_PatInfo+Einwilligung_V02F_2019-11-06_mÄ

Folgende Mitglieder haben an der Beratung des o.g. Antrages mitgewirkt:

Prof. Dr. Stefan Engeli (Vorsitzender)
Stellv. Leiter des Instituts für Klinische Pharmakologie, MHH

PD Dr. Urs-Vito Albrecht (stellv. Vorsitzender & geschäftsführender Arzt)
Stellv. Direktor des Peter L. Reichertz Instituts für Medizinische Informatik, MHH

Prof. Dr. Anibh Martin Das
Leiter der Pädiatrischen Stoffwechselmedizin, Klinik für Pädiatrische Nieren-, Leber- und Stoffwechselerkrankungen, MHH

Dr. Justus Graubner
Arzt für Allgemeinmedizin

Prof. Dr. Armin Koch
Leiter des Instituts für Biometrie, MHH

Frau Prof. Dr. Brigitte Lohff
Em. Leiterin des Instituts für Geschichte, Ethik und Philosophie der Medizin, MHH

Dr. Oliver Pramann
Rechtsanwalt und Notar, Fachanwalt für Medizinrecht

Dipl.-Ing. Jörg Viering
Komm. Leiter der Zentralen Forschungswerkstätten, MHH

Prof. Dr. Peter M. Vogt
Direktor der Klinik für Plastische, Ästhetische, Hand- & Wiederherstellungschirurgie, MHH

Beratung bei Fragen zur Genetik und zu Biobanken:

Prof. Dr. Thomas Illig
Leiter der Hannover Unified Biobank (HUB) & Stellv. Direktor des Instituts für Humangenetik, MHH
