# Supplementary material for: Viral load-guided immunosuppression after lung transplantation (VIGILung)—study protocol for a randomized controlled trial
Source: Trials. 2021 Jan 11;22:48. doi: 10.1186/s13063-020-04985-w (PMC7798016; doi:10.1186/s13063-020-04985-w)
Supplement: Supplementary file 4 — Additional file 4. Translation of the Ethics committee’s vote of Medical University of Vienna [file 13063_2020_4985_MOESM4_ESM.pdf]

ethik kommission
Medizinische Universität Wien

Borschkegasse 8b/6
1090 Wien, Österreich
T +43(0)1 404 00-21470, 22440
F +43(0)1 404 00-16900
ethik-kom@meduniwien.ac.at
http://ethikkommission.meduniwien.ac.at/

Votum:

EK Nr: 1127/2020
EudraCT Nr: 2019-001770-29
Projekttitel: Viruslast gesteuerte Immunsuppression nach Lungentransplantation Eine offene, randomisierte, kontrollierte, Parallel-Gruppen, multizentrische Studie (VIGILung)
Antragsteller/in: Frau Karin Weide
Institution: Philipps-Universität Marburg, Koordinierungszentrum für klinische Studien (KKS)
Sponsor: Philipps Universität Marburg, Koordinierungszentrum für klinische Studien (KKS)

Teilnehmende Prüfzentren:

Ethik-Kommission	Prüfzentrum	Prüfärztin/arzt
Ethikkommission der Medizinischen Universität Wien	Klinische Abteilung für Thoraxchirurgie, Medizinische Universität Wien	Herr PD Dr. med. Peter Jaksch

Die Stellungnahme der Ethik-Kommission erfolgt aufgrund folgender eingereichter Unterlagen:

Conflict of Interest

Name	Version	Datum
11_Conflict of Interest_PD Dr. Peter Jaksch	V01F	22.05.2019

Covering Letter

Name	Version	Datum
01_2020-02-04_VIGILung_Brief an EK Wien	V01F	04.02.2020
01_2020-05-05_VIGILung_Brief an EK Wien	V01F	05.05.2020
01_2020-05-28_VIGILung_Brief an EK Wien	V01F	28.05.2020

Lebenslauf (CV)

Name	Version	Datum
08_CV_Dr. Hielle-Wittmann_2020-01-13	Januar 2020	13.01.2020
08_CV_Dr. Lambers_2020-01-14	Januar 2020	14.01.2020

Name	Version	Datum
08_CV_Dr. Muraközy_2020-01-14	Januar 2020	14.01.2020
08_CV Dr. Jaksch_2020-01-15	Januar 2020	15.01.2020

EudraCT-Formular

Name	Version	Datum
02_Application for EudraCT Number	V01F	15.04.2019
02_VIGILung_V01F_2019-001770-29 AT 20200204 CTA PDF Form_EK_mU	V01F	04.02.2020
2_VIGILung_V02F_2019-001770-29 AT 20200304 CTA PDF Form_EK_mU	V02F	04.03.2020

Versicherungsbestätigung

Name	Version	Datum
4B_2020-01-31_VIGILung_Versicherungsschein_Unfall_ Wien	V01F	31.01.2020
4B_VIGILung_Wegeunfallversicherung_VB_SVAUB 2017	V01F	31.01.2020
4B_VIGILung_Wegeunfallversicherung_VB_ZB Gruppen-Unfall	V01F	31.01.2020
4A_2020-03-02_VIGILung_Police_Probanden_Wien	V01F	02.03.2020
4A_VIGILung_Versicherungsbedingungen_E__4u_Wien	V01F	02.03.2020
03A_2020-05-27_VIGILung_Bestätigung_Probanden_Wien	V01F	27.05.2020
03B_2020-05-27_VIGILung_Police_Probanden_Wien	V01F	27.05.2020

Prüferinformation (Investigator's Brochure)

Name	Version	Datum
06_Fachinformation-DecortinH(Prednisolon)-September-2017	September 2017	01.09.2017
06_Fachinformation-Myfortic(Mycophenolat-Natrium)-August-2018	August 2018	01.08.2018
06_Fachinformation-Prograf(Tacrolimus)-September-2019	September 2019	01.09.2019
06_Fachinformation-CellCept(Mycophenolat-Mofetil)-Oktober-2019	Oktober 2019	01.10.2019
06_Fachinformation-Imurek(Azathioprin)-November-2019	November 2019	01.11.2019

Sonstige

Name	Version	Datum
12_VIGILUNG Weiterleitungsvereinbarung Wien V02 D01 2019-09-19_geprüft	V02D01	19.09.2019
10_2019-11-19_VIGILung_positives Votum EK MHH	V01F	19.11.2019
10_2020-01-24_VIGILung_Amendment Nr.1 pos. Votum EK Hannover	V01F	24.01.2020
09_2020-01-30_VIGILung_Liste der Zentren_Ethikkommissionen_Behörden_V01F	V01F	30.01.2020
05_2020-04-29_Bestaetigung AMG 2019-001770-29	V01F	29.04.2020

Patienteninformation

Name	Version	Datum
04_VIGILung_PatInfo+Einwilligung_V02F_2019-11-06	V02F	06.11.2019
04_VIGILung_PatInfo +Einwilligung_V02F_2019-11-06_Wien	V02F_Wien	06.11.2019
3A_VIGILung_PatInfo +Einwilligung_V03F_2020-05-05_Wien_mÄ	V03F	05.05.2020
3B_VIGILung_PatInfo +Einwilligung_V03F_2020-05-05_Wien	V03F	05.05.2020
02A_VIGILung_PatInfo +Einwilligung_V04F_2020-05-28_Wien_mÄ	V04F	28.05.2020
02B_VIGILung_PatInfo +Einwilligung_V04F_2020-05-28_Wien	V04F	28.05.2020

Studienprotokoll (Prüfplan)

Name	Version	Datum
03_VIGILung_Study-Protocol_Unterschriften_V03F_2019-11-05	V03F	05.11.2019
03_VIGILung_Study-Protocol_V03F_2019-11-05	V03F	05.11.2019

Die Kommission fasst folgenden Beschluss (mit X markiert):

X	Es besteht kein Einwand gegen die Durchführung der Studie.<br><br>ACHTUNG: Unter Berücksichtigung der "ICH-Guideline for Good Clinical Practice" gilt dieser Beschluss ein Jahr ab Datum der Ausstellung. Gegebenenfalls hat der Antragsteller eine Verlängerung der Gültigkeit rechtzeitig zu beantragen.

Ergänzende Kommentare der Sitzung am 03.03.2020:
Zum Antrag:
Punkt 6.2: Rein studienbedingte Maßnahmen sind ggf. im Detail anzuführen.

Zur Teilnehmerinformation:
Punkt 3: Es sollte deutlicher hervorgehen, ob die angeführten Untersuchungen nur bei Studienteilnahme erfolgen oder es sich um die auch in der üblichen Routine erfolgenden Maßnahmen und Studienbesuche handelt. Allfällige rein für die Studie erfolgende Maßnahmen (z.B. Abnahme zusätzlicher Blutmengen, zusätzliche Fragebögen, zusätzliche Besuche am Studienzentrum) sollten deutlich als solche gekennzeichnet sein. Beim 6-Minuten Gehtest sollte es anstatt "in 6 Minuten laufen können" heißen "in 6 Minuten gehen können" (kommt mehrmals vor).
Punkt 5: Sollte es Maßnahmen geben, die nur für die Studie erfolgen (siehe oben), so sind deren Risiken und Unannehmlichkeiten ggf. anzuführen. Der letzte Satz sollte gestrichen werden, die Belastungen und Risiken durch die immunsuppressiven Medikamente sind nicht durch die Studienteilnahme bedingt. Es ist allerdings zu diskutieren, ob die Zuteilung zur Studiengruppe potentiell mit zusätzlichen Risiken verbunden sein kann.
Punkt 7: Die Ethik-Kommission weist darauf hin, dass gemäß dem österreichischen AMG bei gebärfähigen Frauen in monatlicher Wiederholung während der klinischen Prüfung ein Schwangerschaftstest durchgeführt werden muss. Der Text und die Vorgangsweise sind anzupassen.
Punkt 8: Es ist zu ergänzen, dass auch keine Vergütung erfolgt.
Punkt 9 ist zu ersetzen durch den Versicherungspassus aus der Musterinformation für klinische Prüfungen. Die Versicherungsdaten sind zu ergänzen.
Punkt 12: Es wird ersucht, den Text durch den Datenschutzpassus der aktuellen Musterinformation zu ersetzen (zu finden auf der Website der Ethik-Kommission unter http://ethikkommission.meduniwien.ac.at/service/patienteninformation/). Dieser wurde in Übereinstimmung mit dem Forum der österreichischen Ethik-Kommissionen und Datenschutzexperten erstellt und ist seit 5.6.2019 verpflichtend zu verwenden. Allfällige Ergänzungen zum Datenschutz können ausschließlich am Ende dieses Textabschnitts angefügt werden, wobei darauf zu achten ist, dass es dadurch nicht zu redundanten oder widersprüchlichen Aussagen kommt.
Punkt 13: Bezüglich der im letzten Satz angeführten Biobank ist der lokal zutreffende Aufbewahrungsort anzugeben. Auch sind bezüglich der Biobank folgende Informationen zu ergänzen: Es ist anzuführen, wie lange die Proben gelagert werden und wer (Person oder Funktionsinhaber) für Lagerung und Vernichtung der Proben verantwortlich ist, und es muss grob eingeschränkt werden, zu welchem Forschungsgebiet die Proben zukünftig untersucht werden dürfen. Es muss weiters erwähnt werden, dass jedes neue Forschungsprojekt an den aufbewahrten Proben zuvor einer Ethikkommission vorgelegt wird und dass die Teilnehmer jederzeit ihre Zustimmung rückgängig machen und verlangen können, dass ihre Proben vernichtet werden.
Punkt 14: Es ist sicherzustellen, dass für das lokale Prüfzentrum auch eine 24 Stunden erreichbare Telefonnummer angegeben ist (z.B. Bettenstation, Notfallhandy,...). Diese sollte auch entsprechend gekennzeichnet sein. Kontaktdaten der Patientenanwaltschaft (WPPA) sind anzuführen (Adresse, Telefonnummer, E-Mail).

Seite 8-12: Auch für die Einwilligungserklärung ist der entsprechende Textabschnitt der aktuellen Musterinformation zu verwenden. Die Abschnitte "Datenschutz" und "Information gemäß DSGVO" sind vollständig zu streichen, sämtliche Angaben zum Datenschutz müssen sich in Punkt 12 finden, eine Wiederholung im Einwilligungsteil ist nicht erforderlich.
Seite 13ff: Der Anhang ist vollständig zu streichen, die Belastungen und Risiken durch Tacrolimus sind nicht durch die Studienteilnahme bedingt.

Zur Versicherung: muss nachgereicht werden
Sollte es sich nicht um die Rahmenversicherung der MedUni Wien handeln, sind zusätzlich die Allgemeinen Versicherungsbedingungen für die Personenschadenversicherung (APVA, BPV-A, o.ä genannt), sowie allfällige weitere dem Versicherungsvertrag zugrunde liegende besondere Versicherungsbedingungen vorzulegen.

Andere:
Das unterschriebene Antragsformular ist in Papierform nachzureichen.

Die Ethik-Kommission ersucht die Antragsteller, bei der Wiedervorlage von geänderten Unterlagen ein Exemplar mit hervorgehobenen Änderungen beizulegen.

Ergänzende Kommentare:
Nachtrag vom 23. Juni 2020:
Die Antragsteller legen am 16.06.2020 gültige Versicherungsdokumente (Versicherungsschutz für 72 Studienteilnehmer) sowie überarbeitete Unterlagen vor, die von der Ethik-Kommission akzeptiert werden. Das unterschriebene Antragsformular wurde nachgereicht.
Begründung der Befürwortung des Antrages durch die Ethik-Kommission: Es handelt sich bei dem vorliegenden Projekt um eine relevante Fragestellung, die mit geeigneter Methodik beantwortet werden soll. Die vorgenommene Bewertung des Nutzen/Risikoverhältnisses wird von der Ethik-Kommission anerkannt.
Die Antragsteller werden darauf hingewiesen, dass im Falle von Protokolländerungen die Versicherung zu informieren ist. Bei Fehlen einer automatischen Verlängerung des Versicherungsvertrages ist rechtzeitig eine Versicherungsbestätigung über die Verlängerung vorzulegen.

Die Ethik-Kommission weist darauf hin, dass die behördlich vorgeschriebenen Maßnahmen hinsichtlich der COVID-19 Pandemie beachtet werden müssen. Der Prüfer und der Sponsor müssen in ihrem jeweiligen Wirkungskreis unter allfälliger Beachtung von Leitlinien gewährleisten, dass keine zur Bekämpfung der Pandemie benötigten Ressourcen gebunden werden bzw. ausreichend Personal vorhanden ist und die Teilnehmer durch ihre Studienteilnahme keiner zusätzlichen Infektionsgefahr ausgesetzt werden.

Die Ethik-Kommission geht - rechtlich unverbindlich - davon aus, dass es sich um eine klinische Prüfung gemäß AMG handelt.

Die aktuelle Mitgliederliste der Ethik-Kommission ist unter folgender Adresse abrufbar:
http://ethikkommission.meduniwien.ac.at/ethik-kommission/mitglieder/

Mitglieder der Ethik-Kommission, die für diesen Tagesordnungspunkt als befangen anzusehen waren und daher laut Geschäftsordnung an der Entscheidungsfindung/Abstimmung nicht teilgenommen haben: **keine**

Dieses Dokument ist für berechtigte Benutzer/innen in digitaler Form unter folgender Adresse abrufbar:
https://ekmeduniwien.at/vote/19586/download/

SIGNATURINFORMATIONEN	Unterzeichner	Dr. Jürgen Zezula
	Datum/Zeit-UTC	2020-06-23T13:03:07Z
	Prüfinformation	Informationen zur Prüfung der elektronischen Signatur finden Sie unter: https://www.signaturpruefung.gv.at
