# Supplementary material for: Viral load-guided immunosuppression after lung transplantation (VIGILung)—study protocol for a randomized controlled trial
Source: Trials. 2021 Jan 11;22:48. doi: 10.1186/s13063-020-04985-w (PMC7798016; doi:10.1186/s13063-020-04985-w)
Supplement: Supplementary file 5 — Additional file 5. Tabular view from registration at clinicaltrials.gov concerning WHO trial dataset [file 13063_2020_4985_MOESM5_ESM.pdf]

COVID-19 is an emerging, rapidly evolving situation.
Get the latest public health information from CDC: https://www.coronavirus.gov.
Get the latest research information from NIH: https://www.nih.gov/coronavirus.



# Viral Load Guided Immunosuppression After Lung Transplantation (VIGILung)

The safety and scientific validity of this study is the responsibility of the study sponsor and investigators. Listing a study does not mean it has been evaluated by the U.S. Federal Government. Know the risks and potential benefits of clinical studies and talk to your health care provider before participating. Read our disclaimer for details.

ClinicalTrials.gov Identifier: NCT04198506

Recruitment Status : Recruiting
First Posted : December 13, 2019
Last Update Posted : September 17, 2020

See Contacts and Locations

**Sponsor:**
Philipps University Marburg Medical Center

**Information provided by (Responsible Party):**
Philipps University Marburg Medical Center



## Tracking Information

| | |
|---|---|
| **First Submitted Date** ICMJE | December 5, 2019 |
| **First Posted Date** ICMJE | December 13, 2019 |
| **Last Update Posted Date** | September 17, 2020 |
| **Actual Study Start Date** ICMJE | August 5, 2020 |
| **Estimated Primary Completion Date** | December 1, 2025 (Final data collection date for primary outcome measure) |
| **Current Primary Outcome Measures** ICMJE **(submitted: December 12, 2019)** | ΔGFR change of the glomerular filtration rate GFR [ Time Frame: Between randomization and 12 months thereafter ]<br>The primary efficacy endpoint ΔGFR is defined as the change of the glomerular filtration rate GFR between randomization and 12 months thereafter. GFR will be estimated using the CKD-EPI formula. |

| | |
|---|---|
| **Original Primary Outcome Measures** ICMJE | *Same as current* |
| **Change History** | Complete list of historical versions of study NCT04198506 on ClinicalTrials.gov Archive Site |
| **Current Secondary Outcome Measures** ICMJE **(submitted: January 27, 2020)** | • GFR (CKD-EPI) [ Time Frame: 1 and 2 months after transplantation (screening visits) and 0, 3, 6, 9 and 12 months after randomization ]<br>Glomerular filtration rate (the Chronic Kidney Disease Epidemiology Collaboration - CKD-EPI) formula<br>• GFR (Cystatin) [ Time Frame: Screening visits and 0, 3, 6, 9 and 12 months after randomization ]<br>Glomerular filtration rate (Cystatin)<br>• Number of biopsy-proven acute cellular rejection (grade A1 or higher) [ Time Frame: 12 months after randomization ]<br>• Number of episodes of biopsy-proven lymphocytic bronchitis (grade B1R or higher) [ Time Frame: 12 months after randomization ]<br>• Number of cytomegalovirus (CMV)-infections and CMV-disease episodes [ Time Frame: 12 months after randomization ]<br>• Number of community-acquired respiratory viral infections (CARV) [ Time Frame: 12 months after randomization ]<br>• Number of fungal and bacterial infections [ Time Frame: 12 months after randomization ]<br>• Number of unscheduled or emergency hospitalizations [ Time Frame: 12 months after randomization ]<br>• Number of ICU admissions [ Time Frame: 12 months after randomization ]<br>• Quality of life questionnaire [ Time Frame: Screening visits and 0, 3, 6, 9 and 12 months after randomization ]<br>European Quality of Life 5 Dimensions - EQ-5D<br>• New or progressive malignancy [ Time Frame: 12 months after randomization ]<br>• Median tacrolimus trough levels [ Time Frame: Screening visits and 0, 3, 6, 9 and 12 months after randomization ]<br>• Tacrolimus dose [ Time Frame: Screening visits and 0, 3, 6, 9 and 12 months after randomization ]<br>• Number of changes (increase or decrease) in target trough levels of tacrolimus [ Time Frame: Screening visits and 0, 3, 6, 9 and 12 months after randomization ]<br>• Exercise capacity (6-Min Walk Test - 6MWT) [ Time Frame: At randomization and 12 months thereafter ]<br>• CD4-Lymphocytes counts [ Time Frame: 0, 6 and 12 months after randomization ]<br>• Donor specific antibodies [ Time Frame: 0, 6 and 12 months after randomization ]<br>• FEV1 in % best value [ Time Frame: Screening visits and 0, 3, 6, 9 and 12 months after randomization ]<br>• Incidence of chronic lung allograft dysfunction [ Time Frame: Between randomization and 12 months thereafter ]<br>• IgG-level [ Time Frame: 0, 6 and 12 months after randomization ]<br>• Use of rescue immunotherapy (defined by the use of ATG, Rituximab, Alemtuzumab, plasma exchange, immunoadsorption) [ Time Frame: 12 months after |

| | |
|---|---|
| | randomization ]<br>• Death or re-do transplantation [ Time Frame: 12 months after randomization ] |
| **Original Secondary Outcome Measures** ICMJE **(submitted: December 12, 2019)** | • GFR (CKD-EPI) [ Time Frame: 1 and 2 months after transplantation (screening visits) and 0, 3, 6, 9 and 12 months after randomization ]<br>Glomerular filtration rate (the Chronic Kidney Disease Epidemiology Collaboration - CKD-EPI) formula<br>• GFR (Cystatin) [ Time Frame: Screening visits and 0, 3, 6, 9 and 12 months after randomization ]<br>Glomerular filtration rate (Cystatin)<br>• Number of biopsy-proven acute cellular rejection (grade A1 or higher) [ Time Frame: 12 months after randomization ]<br>• Number of cytomegalovirus (CMV)-infections and CMV-disease episodes [ Time Frame: 12 months after randomization ]<br>• Number of community-acquired respiratory viral infections (CARV) [ Time Frame: 12 months after randomization ]<br>• Number of fungal and bacterial infections [ Time Frame: 12 months after randomization ]<br>• Number of unscheduled or emergency hospitalizations [ Time Frame: 12 months after randomization ]<br>• Number of ICU admissions [ Time Frame: 12 months after randomization ]<br>• Quality of life [ Time Frame: Screening visits and 0, 3, 6, 9 and 12 months after randomization ]<br>European Quality of Life 5 Dimensions - EQ-5D<br>• New or progressive malignancy [ Time Frame: 12 months after randomization ]<br>• Median tacrolimus trough levels [ Time Frame: Screening visits and 0, 3, 6, 9 and 12 months after randomization ]<br>• Tacrolimus dose [ Time Frame: Screening visits and 0, 3, 6, 9 and 12 months after randomization ]<br>• Number of changes (increase or decrease) in target trough levels of tacrolimus [ Time Frame: Screening visits and 0, 3, 6, 9 and 12 months after randomization ]<br>• Exercise capacity (6-Min Walk Test - 6MWT) [ Time Frame: At randomization and 12 months thereafter ]<br>• CD4-Lymphocytes counts [ Time Frame: 0, 6 and 12 months after randomization ]<br>• Donor specific antibodies [ Time Frame: 0, 6 and 12 months after randomization ]<br>• FEV1 in % best value [ Time Frame: Screening visits and 0, 3, 6, 9 and 12 months after randomization ]<br>• Incidence of chronic lung allograft dysfunction [ Time Frame: Between randomization and 12 months thereafter ]<br>• IgG-level [ Time Frame: 0, 6 and 12 months after randomization ]<br>• Use of rescue immunotherapy (defined by the use of ATG, Rituximab, Alemtuzumab, plasma exchange, immunoadsorption) [ Time Frame: 12 months after randomization ]<br>• Death or re-do transplantation [ Time Frame: 12 months after randomization ] |

| | |
|---|---|
| **Current Other Pre-specified Outcome Measures** | *Not Provided* |
| **Original Other Pre-specified Outcome Measures** | *Not Provided* |

## Descriptive Information

| | |
|---|---|
| **Brief Title** ICMJE | Viral Load Guided Immunosuppression After Lung Transplantation |
| **Official Title** ICMJE | Viral Load Guided Immunosuppression After Lung Transplantation, an Open-label, Randomized, Controlled, Parallel-group, Multicenter Trial |
| **Brief Summary** | The VIGILung study is an open-label, randomized, multicenter trial in lung transplant recipients to investigate the safety and efficacy of personalized immunosuppression guided by DNA monitoring of Torque-Teno-Virus (TTV). The aim of the study is to investigate an individual adaptation of the calcineurin inhibitor tacrolimus (tailored calcineurin inhibitor dosing) by a non-invasive biomarker (TTV viral load in whole blood) compared to conventional calcineurin inhibitor dosing. Indicator for toxicity will be the glomerular filtration rate (GFR), which will be estimated using the CKD-EPI formula. 250 patients (age ≥ 18 years) with 21 to 42 days after de novo lung transplantation (bilateral or combined) will be screened as possible subjects eligible for the study. N = 144 patients have to be randomized in two study arms. In Arm 1 tacrolimus doses will be adapted according to the tacrolimus blood level (conventional therapeutic drug monitoring - TDM) and additionally depending on TTV viral load. In Arm 2 tacrolimus doses will be adapted according to TDM. |
| **Detailed Description** | *Not Provided* |
| **Study Type** ICMJE | Interventional |
| **Study Phase** ICMJE | Not Applicable |
| **Study Design** ICMJE | Allocation: Randomized<br>Intervention Model: Parallel Assignment<br>Masking: None (Open Label)<br>Primary Purpose: Treatment |
| **Condition** ICMJE | Transplantation Lung |
| **Intervention** ICMJE | • Other: Tailored tacrolimus dosing<br>Tacrolimus doses will be adapted according to tacrolimus blood level (conventional therapeutic drug monitoring -TDM) and additionally depending on TTV viral load.<br>• Other: Conventional tacrolimus dosing<br>Tacrolimus doses will be adapted according to tacrolimus blood level (conventional therapeutic drug monitoring - TDM). |

| | |
|---|---|
| **Study Arms** ICMJE | - Experimental: Tailored tacrolimus dosing<br>Tacrolimus doses will be adapted according to tacrolimus blood level (conventional therapeutic drug monitoring - TDM) and additionally depending on TTV viral load.<br>Intervention: Other: Tailored tacrolimus dosing<br>- Active Comparator: Conventional tacrolimus dosing<br>Tacrolimus doses will be adapted according to tacrolimus blood level (conventional therapeutic drug monitoring - TDM).<br>Intervention: Other: Conventional tacrolimus dosing |
| **Publications *** | *Not Provided* |

*** Includes publications given by the data provider as well as publications identified by ClinicalTrials.gov Identifier (NCT Number) in Medline.**

## Recruitment Information

| | |
|---|---|
| **Recruitment Status** ICMJE | Recruiting |
| **Estimated Enrollment** ICMJE **(submitted: December 12, 2019)** | 144 |
| **Original Estimated Enrollment** ICMJE | *Same as current* |
| **Estimated Study Completion Date** ICMJE | December 1, 2025 |
| **Estimated Primary Completion Date** | December 1, 2025 (Final data collection date for primary outcome measure) |
| **Eligibility Criteria** ICMJE | Inclusion Criteria:<br>1. Patients 21 to 42 days after de novo lung transplantation (bilateral or combined)<br>2. Age ≥ 18 years<br>3. Tacrolimus based immunosuppression<br>4. Written informed consent<br>5. Detectable TTV load at randomization (>2,7 log 10)<br>6. Negative serum pregnancy test in women of childbearing potential<br>7. Women of childbearing capacity must agree to maintain highly effective methods of contraception by practicing abstinence or by using at least two methods of birth control from the date of consent through the end of the study. If abstinence is not practiced, a combination of hormonal contraceptive (oral, injectable or implants) and a barrier method (condom, diaphragm with a vaginal spermicidal agent) has to be used<br>Exclusion Criteria:<br>1. History or high-risk of obstructive airway complications after lung transplantation<br>2. Respiratory failure (need for oxygen therapy or ventilation at screening after lung transplantation)<br>3. Inability to undergo transbronchial biopsy<br>4. Advanced kidney failure (GFR CKD-EPI <30 ml/min/1.73m²) at inclusion and/or current renal replacement therapy at inclusion or randomization |

| | |
|---|---|
| | 5. Advanced liver cirrhosis (CHILD-Pugh Score C) after lung transplantation<br>6. Fluctuating tacrolimus drug levels (less than 20% in target range after transplantation)<br>7. Symptoms of significant mental illness and with inability to cooperate or communicate with the investigator<br>8. Unlikeliness to comply with the study requirements<br>9. HIV positivity<br>10. Evidence of unsolved drug or alcohol addiction<br>11. Breastfeeding women<br>12. Simultaneous participation in other clinical trials if not permitted by the steering committee |
| **Sex/Gender** ICMJE | Sexes Eligible for Study: All |
| **Ages** ICMJE | 18 Years and older (Adult, Older Adult) |
| **Accepts Healthy Volunteers** ICMJE | No |
| **Contacts** ICMJE | Contact: Jens Gottlieb, Prof. MD +49 (0) 511-532-4601 gottlieb.jens@mh-hannover.de |
| **Listed Location Countries** ICMJE | Austria, Germany |
| **Removed Location Countries** | |

## Administrative Information

| | |
|---|---|
| **NCT Number** ICMJE | NCT04198506 |
| **Other Study ID Numbers** ICMJE | KKS-256<br>2019-001770-29 ( EudraCT Number ) |
| **Has Data Monitoring Committee** | Yes |
| **U.S. FDA-regulated Product** | Studies a U.S. FDA-regulated Drug Product: No<br>Studies a U.S. FDA-regulated Device Product: No |
| **IPD Sharing Statement** ICMJE | *Not Provided* |
| **Responsible Party** | Philipps University Marburg Medical Center |
| **Study Sponsor** ICMJE | Philipps University Marburg Medical Center |
| **Collaborators** ICMJE | *Not Provided* |
| **Investigators** ICMJE | Principal Investigator: Jens Gottlieb, Prof. MD — Klinik für Pneumologie OE 6870, Medizinische Hochschule Hannover (MHH) |
| **PRS Account** | Philipps University Marburg Medical Center |
| **Verification Date** | September 2020 |

ICMJE **Data element required by the International Committee of Medical Journal Editors and the World Health Organization ICTRP**
